# Cell division cycle protein 42-driven activation of the MKK3/6-p38 signaling pathway participates in cardiac remodeling in mice

**DOI:** 10.1007/s00018-025-05743-4

**Published:** 2025-07-03

**Authors:** Ke Wen, Lin Xie, Quan-Wen Liu, Guan-Hui Yu, Xu-Hui Qiao, Yu-Chun Huang, Lu Wang, Xin Li, Li-Dan Wen, Xiao-Lei Wang, Jing He, Xin-Yu Xiao, Xiao-Xiao Zhao, Ling-Fang Wang, Hong-Bo Xin, Ke-Yu Deng

**Affiliations:** 1 The National Engineering Research Center for Bioengineering Drugs and the Technologies, Institute of Translational Medicine, Jiangxi Medical College, Nanchang University, 330031 Nanchang, China; 2 School of Pharmacy, Jiangxi Medical College, Nanchang University, 330031 Nanchang, China; 3 School of Life and Science, Nanchang University, Nanchang, 330031 China; 4 Discipline of Chinese and Western Integrative Medicine, Jiangxi University of Traditional Chinese Medicine, 330004 Nanchang, China; 5 Jiangxi Provincial People’s Hospital, The First Affiliated Hospital of Nanchang Medical College, 330006 Nanchang, China

**Keywords:** Cardiac function, Cardiomyocyte hypertrophy, Apoptosis, Inflammation

## Abstract

Cell division cycle protein 42 (Cdc42) is a member of the Rho GTPase subfamily that serves as a signal mediating factor in cell cycle division, cytoskeleton arrangement, cell polarization, membrane trafficking and signal transduction. However, the role of Cdc42 in cardiac remodeling, including hypertrophy and fibrosis, remains controversial. This study aimed to clarify the role and underlying mechanism of Cdc42 in cardiac remodeling. Cardiac Cdc42 knockout (Cdc42^*CKO*^) mice were generated by crossing Cdc42^*loxP/loxP*^ mice with MLC2v-Cre mice. Mouse cardiac remodeling models were induced by subcutaneous administration of AngII (1500 ng/kg/min) for 7 days or transverse aortic constriction (TAC) for 2 or 8 weeks. Our results showed that cardiac Cdc42 deletion significantly suppressed AngII- or TAC-induced cardiac hypertrophy and fibrosis and improved cardiac function in mice. Cdc42^*CKO*^ or specific inhibition of Cdc42, markedly inhibited Ang II-mediated activation of the MKK3/6-p38 cascade in the heart and in isolated newborn/adult mouse cardiomyocytes or H9c2 cells. Furthermore, Cdc42 overexpression increased the surface area and hypertrophic gene expression in myocytes, whereas ML141 (a Cdc42 inhibitor) and SB203580 (a p38 inhibitor) specifically decreased p38 activation and hypertrophy in Cdc42-overexpressing or AngII-induced hypertrophic cardiomyocytes, indicating that p38 is a downstream effector of Cdc42 in cardiac hypertrophy. Taken together, our results demonstrated that Cdc42 is a key driver of cardiac remodeling via activation of the p38 signaling pathway.

**Graphical abstract:**

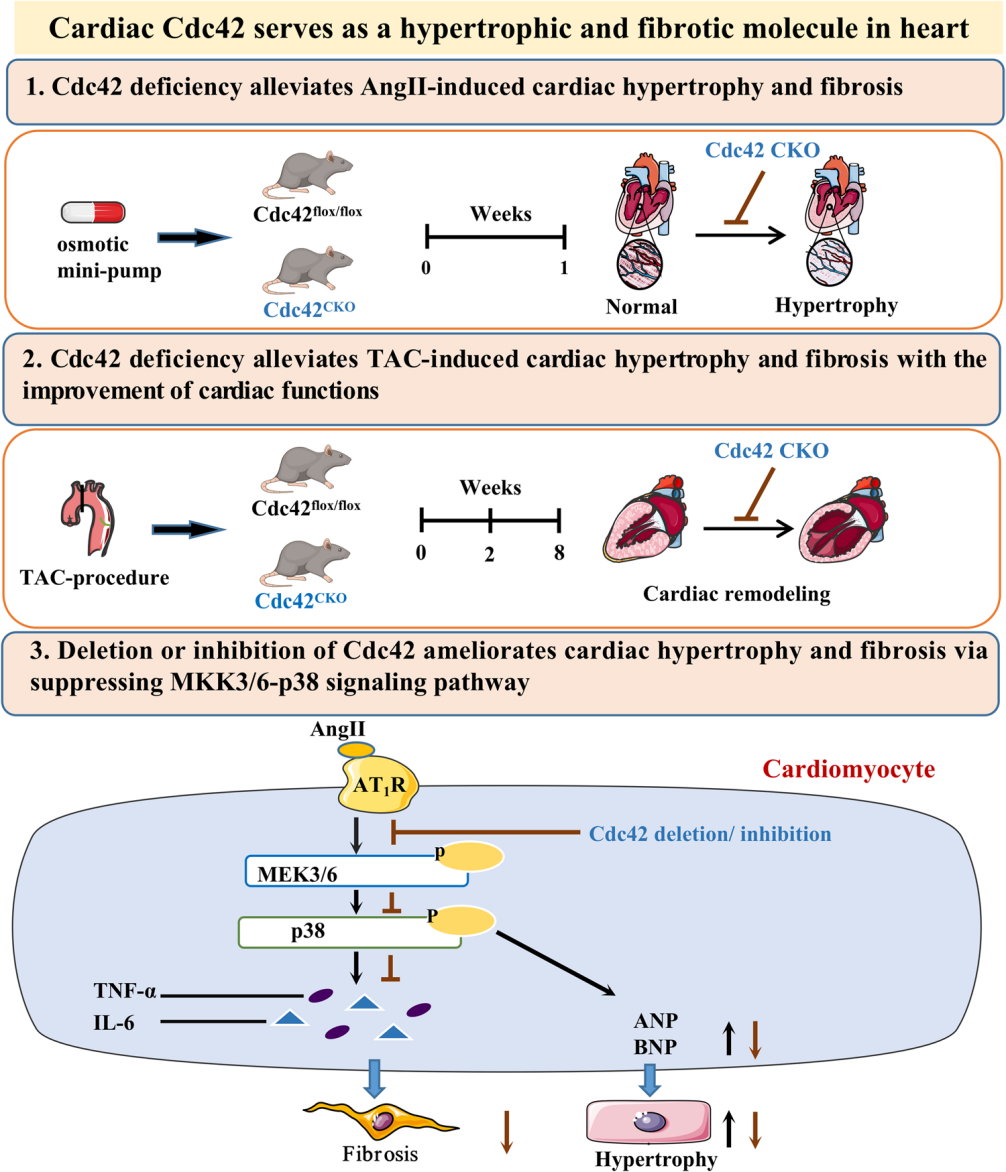

**Supplementary Information:**

The online version contains supplementary material available at 10.1007/s00018-025-05743-4.

## Highlights

- Cell division cycle protein 42 (Cdc42) participates in cell cycle division, cytoskeleton arrangement, cell polarization, membrane trafficking and signal transduction.
- Cardiac deletion of Cdc42 protects against multiple mouse models of cardiac remodeling, including hypertrophy and fibrosis, both *in vivo* and *in vitro*.
- Cardiac Cdc42 deficiency attenuates cardiac remodeling by suppressing hypertrophy, apoptosis and inflammation via inhibiting MKK3/6-p38 signaling pathway in myocardium.

**Supplementary Information:**

The online version contains supplementary material available at 10.1007/s00018-025-05743-4.

## Introduction

Cardiac remodeling, including pathological hypertrophy and fibrosis, is an adaptive response to cardiac stress mostly due to hypertension at the initial point, which eventually progresses to heart failure, arrhythmia or stroke over time [1]. The activation of the MAPK signaling pathway plays an important role in cardiac hypertrophy [2], and the activation of mitogen-activated protein kinase p38 (p38-MAPK) is associated with multiple stimuli, such as angiotensin II (Ang II), endothelin 1 or catecholamine-induced cardiac hypertrophy and fibrosis [3], whereas the inhibition of p38-MAPK improves vascular function in the aorta and kidneys of Ang II-treated mice [4].

Cell division cycle protein 42 (Cdc42) is a member of the Rho GTPase subfamily of small GTP-binding proteins, in which Cdc42 is widely expressed in a variety of cell types and serves as a molecular switch in cell cycle division, cytoskeleton arrangement, cell polarization, membrane trafficking and signal transduction [5]. Cdc42 has been implicated in the activation of ERK, JNK and p38-MAPK [6, 7] and cooperates with Raf to activate ERK and induce transformation, such as by regulating cytoskeletal organization and cell shape in dividing cells, indicating that Cdc42 plays a role in regulating the p38 cascade [8]. In addition, Cdc42 is activated by FAK to increase cytoskeletal organization, thus promoting cell contraction [9]. Our previous experimental results showed that pancreatic beta cell-specific deletion of Cdc42 decreased the expression and secretion of insulin by inhibiting the Erk-NeuroD signaling pathway [10]. Cdc42 plays important roles in cardiomyocyte proliferation and heart development, and inactivation of Cdc42 in embryonic cardiomyocytes results in retarded heart development [11]. Cdc42 has been reported to be involved in cardiac hypertrophy both in vivo and in vitro [12–14], however, the role of Cdc42 in cardiac hypertrophy and fibrosis remains unclear or controversial [15]. Therefore, further clarification of the roles and mechanisms of Cdc42 in cardiac remodeling is warranted.

In the present study, cardiomyocyte-specific Cdc42 knockout mice were generated by crossing Cdc42-loxP mice with Cre recombinase knock-in mice driven by the endogenous MLC2v promoter. Cardiomyocyte-specific deletion of Cdc42 significantly alleviated transverse aortic constriction (TAC)- and AngII-induced cardiac hypertrophy and fibrosis. In addition, overexpression of Cdc42 or Ang II markedly increased the myocyte surface area and the expression of hypertrophic genes in vitro. Furthermore, we demonstrated that Cdc42 deficiency or inhibition attenuated cardiac remodeling via inhibition of the MEKK3/6-p38 cascade.

## Materials and methods

### Animal models

The animal experiments were performed according to the Guidelines for the Care and Use of Laboratory Animals in China and were approved by the Animal Ethics Committee of Nanchang University. Cardiomyocyte-specific Cdc42 conditional knockout (Cdc42^*CKO*^) mice were generated by crossing Cdc42^*loxP/loxP*^ mice with MLC2v-Cre mice [16–18], and all the mice were backcrossed to the C57BL/6 background for at least 6 generations before the experiments. The mice were housed in individually ventilated cages under standard conditions with a 12-hour light/dark cycle and were given access to a rodent chow diet (Beijing Keao Xieli Feed Co., Ltd.) and autoclaved water ad libitum. The mice were euthanized via carbon dioxide (CO_2_) inhalation or by cervical dislocation after being anesthetized via the inhalant isofluorane as needed. Cardiac hypertrophy was induced in 2-month-old male mice by AngII (1500 ng/kg/min, Alzet, Cat. No. 2002) infusion with a 7-day osmotic mini-pump (Alzet, model 1007D, Cupertino, CA) or saline alone as described [19, 20]. Cdc42^*CKO*^ and Cdc42^*loxP/loxP*^ mice were divided into AngII–treated and saline groups, respectively. The systolic/diastolic blood pressure and pulse were measured in conscious mice by a tail-cuff system (BP-98 A; Softron, Tokyo, Japan) on day 0 and day 7 post-implanting minipump, and the average of ten consecutive measurements was recorded.

Minimally invasive transverse aortic constriction (mTAC) surgery without chest opening was performed according to a previous protocol with modifications in mice [21]. Briefly, mice were anesthetized via inhalation of 2–4% isoflurane mixed with air, fixed in the supine position on a warm anatomical plate, and maintained unconsciously by endotracheal intubation at 120–130 breaths/minute and a tidal volume of 1–3 ml with isoflurane by a rodent ventilator (ALC-VBS). mTAC surgery was performed around the area between the second and third ribs on the left side to expose the thymus, the thymus lubes were carefully separated from one another, and the aorta was visually exposed under a dissecting microscope. The aortic arch was slightly lifted with a blunt needle parallel to a 27-gauge cushion needle, and the transverse aorta at the site between the right and left common carotid artery was tightened with a 6 − 0 nonabsorptive silk thread. After the animals regained self-breathing and consciousness, they were monitored in individually ventilated cages for 48 h for postoperative signs. Echocardiography was used to evaluate the success of banding and cardiac morphology and function after 2 and 8 weeks.

### Echocardiography

Echocardiography was performed on mice at AngII infusion day 7, on TAC week 2 or week 8 with a high-resolution Micro-Ultrasound system (Vevo2100 or Vevo3100; Visual Sonics, Toronto, Ontario, Canada), in which the mouse was anesthetized by inhalation of 1.5–2% isoflurane mixed with air during the entire echocardiography procedure. The values of intraventricular septal thickness (IVS), left ventricular internal dimension (LVID) and left ventricle posterior wall thickness (LVPWth) at the diastole and systole stages, LV fractional shortening (FS), and LV ejection fraction (EF) were collected and analyzed.

### Histology

The hearts were isolated, fixed in 4% paraformaldehyde for 24 h and embedded in paraffin. The cross sections were harvested at 5 μm and stained with hematoxylin and eosin (H&E, Solarbio, Cat. No. G1120) for histological analysis or Masson trichrome (Solarbio, Cat. No. G1340) for fibrous areas.

### Isolation and culture of cardiomyocytes from adult mice

Cardiomyocytes were isolated from 8- to 10-week-old Cdc42^*CKO*^ and Cdc42^*loxP/loxP*^ mice as described previously [22, 23]. In brief, the mice were anesthetized by an intraperitoneal injection of an overdose of sodium pentobarbital (150 mg·kg^−1^) with heparin (8,000 U·kg^−1^), and the hearts were rapidly excised into ice-cold perfusion buffer and cannulated *via* the aorta before retrograde perfusion on a modified Langendorff system at 37 °C with filtered calcium-free perfusion buffer containing 113 mM NaCl, 4.7 mM KCl, 0.6 mM KH_2_PO_4_, 0.6 mM Na_2_HPO_4_, 1.2 mM MgSO_4_, 10 mM Na-HEPES, 12 mM NaHCO_3_, 10 mM KHCO_3_, 0.032 mM phenol red, 30 mM taurine, 10 mM BDM, and 5.5 mM glucose at pH 7.0. The hearts were perfused with perfusion buffer at a flow rate of 4 mL/min for approximately 4–5 min until the effluents became clear and then switched to digestion buffer for 10–12 min, which consisted of 50 ml of perfusion buffer with 15,000 U of total type II collagenase (Worthington Biochemical Corporation, Lakewood, NJ, USA, Cat. No. LS0004176) and 50 µM CaCl_2_. When the heart became enlarged, pale, and soft, the heart was gently removed from the cannula, placed in a sterile 60-mm dish containing 2.5 ml of digestion buffer, and gently teased into 10–12 small pieces. The heart pieces and cells were gently pipetted with a Pasteur pipette and transferred into a new 15 ml polypropylene conical tube. The solution was diluted to 10 ml with stop buffer (perfusion buffer with 10% FBS) containing 12.5 µM CaCl_2_. The cells were centrifuged for 3 min at 20 × g, after which the supernatants were removed, and the cell pellets were resuspended in serial stop buffer supplemented with increasing concentrations of Ca^2+^ (100 µmol/L, 400 µmol/L, and 900 µmol/L). Approximately 80% of the cells were rod shaped. Finally, the cells were resuspended in plating medium (43 ml of MEM containing 2 mM L-glutamine, 1.26 mM CaCl2, 5 ml of FBS, 500 mM BDM and 10,000 U/ml penicillin/streptomycin) at 25,000 myocytes/ml. Cardiomyocytes were plated onto 6 cm prelaminated dishes (8 mL/dish) and incubated for 3 h in a 5% CO2 incubator at 37 °C to allow the myocytes to attach. Then, the culture medium (48 ml of MEM containing 2 mM L-glutamine, 1.26 mM CaCl2, 500 mM BDM and 10,000 U/ml penicillin/streptomycin) was changed to the sides. Cardiomyocytes were treated with AngII (1000 ng/ml) or saline for 15 min, respectively.

### Isolation and culture of cardiomyocytes from newborn mice

Neonatal ventricular myocytes were isolated from the hearts of 1- to 3-day-old C57BL/6 mice. Briefly, neonatal hearts were digested with 0.08% trypsin (Gibco, 27250-018) and 0.05% collagenase II (Sigma, C6885) in a shaking water bath (Magnetic Stirrer) at 37 °C and 750 rpm for 10 min 4–5 times and neutralized with 10% FBS in DMEM. Cardiomyocytes were collected and cultured in Dulbecco’s modified Eagle’s medium (DMEM, Gibco, Cat. No. 11965092) supplemented with 10% FBS, penicillin (100 U/mL, Gibco, Cat. No. 15140122) and streptomycin (100 µg/mL, Gibco, Cat. No. 15140122). After preseeding, the isolated cells were placed in an uncoated dish at 37 °C for 90–180 min, and 5-bromo-2-deoxyuridine (BrdU, 100 µmol/L, Sigma, Cat. No. B5002) was added to prevent overgrowth of rapidly proliferating fibroblasts at 37 °C with 5% CO_2_.

### Cell culture

H9c2 cells (ATCC, CRL-1446™) were cultured in DMEM (Thermo Fisher, Wal-tham, MA, USA) supplemented with 10% FBS and 100 µg/mL of each of penicillin and streptomycin (Thermo Fisher, Cat. No. 15140163) at 37 °C with 5% CO_2_. H9c2 cells were treated with AngII (1000 ng/ml) or saline for 15 min, respectively.

### Western blot analysis

Total tissue or cellular proteins were extracted by RIPA buffer containing 25 mmol/L HEPES, pH 7.4, 1% NP40, 137 mmol/L NaCl, 10% glycerol, 50 mmol/L NaF and protease inhibitor cocktail (Roche, Mannheim, Germany). Serum proteins were enriched by precipitating mouse serum with methanol and chloroform [24]. The extracted proteins were separated by 10–15% denaturing SDS‒PAGE and then transferred to NC membranes (Bio-Rad), which were incubated with primary antibodies followed by secondary antibodies. Primary antibodies against Cdc42 (Santa Cruz, sc-8401), RhoA (Abcam, ab187027), ERK1/2 (CST, 4695 S), phospho-ERK1/2 (CST, 4370 S), JNK (Abcam, ab179461), phospho-JNK (Abcam, ab124956), NF-κB/p65 (Abcam, ab16502), phospho-NF-κB/p65 (Abcam, ab86299), CaMKII (Abcam, ab32422), phospho-CaMKII (Millipore, 05–574), NFAT-C4 (Abcam, ab23672), calcineurin (Abcam, ab18932), p38 (Abcam, ab170099), phospho-p38 (Abcam, ab4822), phospho-MKK3/MKK6 (CST, 9231 S), GSK3β (CST, 9315 S), phospho-GSK3β (CST, 9336 S), AKT1 (CST, 2967 S), phospho-AKT1 (CST, 4060 S), PI3 K (CST, 4257 S), phospho-PI3K (CST, 4228 S), IL6 (CST, 12912 S), TNFα (Proteintech, 60298-1-Ig), IL10 (Proteintech, 10008-1-AP), Col1 (Servicebio, GB11013), ANP (Santa Cruz, sc-515701), BNP (Abcam, ab19645), Col3 (Servicebio, GB11015) and GAPDH (Santa Cruz, sc − 47724) were used at 4 °C overnight. Blots were detected with HRP-conjugated goat anti–rabbit or rabbit anti-mouse secondary antibodies (Invitrogen, A16078 for goat anti - rabbit; A28175 for rabbit anti - mouse) for 1 h at room temperature. The luminescence was visualized using a Bio-Rad luminescent imaging system.

### RNA extraction, transcriptome assembly and real-time PCR

Total RNA was extracted from mouse heart tissues by the TRIzol (TaKaRa, Code No. 9109) method. RNA was transcribed into cDNA using random hexamers and oligo (dT) with Superscript III Reverse Transcriptase (Invitrogen, 18080051). The expression levels of Cdc42, Cre and β-Actin were determined using semiquantitative RT‒PCR with the following primer pairs (displayed in order of forward primer, reverse primer and the length of cDNA product, Cdc42: (5′-CGG AGA AGC TGA GGA CAA GA-3′, 5′-TGG GTC CCA ACA AGC AAG AA-3′, 410 bp), Cre: (5′-GAC CAG GTT TCA CTC A-3′, 5′-ACC AGA GTC ATC CTT AGC G-3′, 400 bp) and β-Actin: (5′-ATG GAG GGG AAT ACA GCC C-3′, 5′-TTC TTT GCA GCT CCT TCG TT-3′, 149 bp). Cdc42 and Rac1 mRNA levels were quantified by SYBR Green Real-Time PCR. GAPDH served as an internal control. The reaction mixtures consisted of 10 µL of SYBR Green PCR Master Mix, 8.2 µL of water, 10 ng of DNA, and 0.4 µL of primers (10 mM) using the following primer pairs (forward, reverse): GAPDH (5’-CGT CCC GTA GAC AAA ATG GT-3’, 5’-TTG ATG GCA ACA ATC TCC AC-3’), Cdc42 (5’-GTT GGT GAT GGT GCT GTT-3’, 5’-GGA TAA CTT AGC GGT CGT-3’) and Rac1 (5’- TCT CCA GGA AAT GCA TTG GT-3’, 5’- AGA TGC AGG CCA TCA AGT GT-3’).

Approximately 3 µg of total RNA per mouse heart was used as input material for RNA high-throughput sequencing by the Novogene Experimental Department. Sequencing libraries were generated using the NEBNext^®^ Ultra™ RNA Library Prep Kit for Illumina^®^ (NEB, USA, E7530L) following the manufacturer’s recommendations, and index codes were added to attribute the sequences. Briefly, twelve cDNA libraries were constructed with three samples per group. First-strand cDNA was synthesized using random hexamer primers and M-MuLV reverse transcriptase (RNase H-). Second-strand cDNA synthesis was subsequently performed using DNA polymerase I and RNase H. After adenylation of the 3’ ends of cDNA fragments, NEBNext adaptors with hairpin loop structures were ligated to prepare for hybridization. To preferentially select cDNA fragments 150 ~ 200 bp in length, the library fragments were purified with the AMPure XP system (Beckman Coulter, Beverly, USA, Cat. No. A63880) and selected with USER Enzyme (NEB, USA, Cat. No. M5505L) to obtain adaptor-ligated 150 ~ 200 bp cDNAs, which were subsequently amplified with Phusion High-Fidelity DNA polymerase, universal PCR primers and index (X) primers. Then, the PCR products were purified (AMPure XP system) and assessed on an Agilent Bioanalyzer 2100 system for quality. Clustering of the index-coded samples was performed on a cBot Cluster Generation System using the TruSeq PE Cluster Kit v3-cBot-HS (Illumina) according to the manufacturer’s instructions. After cluster generation, the library preparations were sequenced on an Illumina HiSeq platform, 125 bp/150 bp paired-end reads were generated, and approximately 6 Gb of clean data were obtained per sample. Differential expression analysis (DESeq) was performed after the reads were mapped to the mouse reference genome, followed by GO and KEGG enrichment analysis.

### Immunofluorescence staining

About 5-µm paraffin tissue sections were dewaxed and heated in 1x citrate unmasking solution at 95–98 °C for 10 min, then immersed in 0.25% Triton X-100/PBS (PBST) solution and cooled at RT. The phospho-p38 primary antibody (CST #4511, 1:200) was applied on the sections in antibody dilution buffer and incubated overnight at 4 °C. H9c2 cells grown on glass coverslips were fixed in 4% PFA for 20 min and permeabilized in PBST. The primary antibody (mouse anti-IL-6, CST#12912, 1:100) was applied and incubated overnight at 4 °C. Later, the slides were applied with fluorochome-conjugated secondary antibodies (Proteintech, SA00013-4 or SA00013-1), respectively. Finally, the slides were mounted with anti-fluorescence quenching sealing solution containing DAPI (Santa Cruz, sc-24941). The fluorescent images were obtained under a Zeiss LSM 800 confocal microscope (version ZEN 3.3 software). The mean fluorescence intensity of the sections was analyzed by ImageJ software.

### TUNEL assay

The apoptosis assay was performed according to the manufacturer’s instruction for One-step TUNEL In Situ Apoptosis Kit (Elabscience, E-CK-321). Briefly, the paraffin slides were dewaxed, then incubated in proteinase K 1 mg/ml in PBS solution for 20 min and washed with PBS 3 times. About 50 µL of TUNEL reaction mixture was added per sample and incubated in a dark and wet box at 37 °C for 1 h. After washing 3 times with PBS, an anti-fluorescence sealing solution with DAPI was applied on slide. The fluorescent images were taken under Zeiss LSM 800 confocal microscope.

### Statistical analysis

The data are presented as the mean ± SD. *Student’s t test* was used for two-group analyses. One-way or two-way analysis of variance (ANOVA) was used to compare data among three or more groups. *P* < 0.05 was considered to indicate statistical significance.

## Results

### Cardiomyocyte-specific deletion of Cdc42 protected the heart from AngII-induced cardiac hypertrophy and fibrosis in mice

**Fig. 1:**
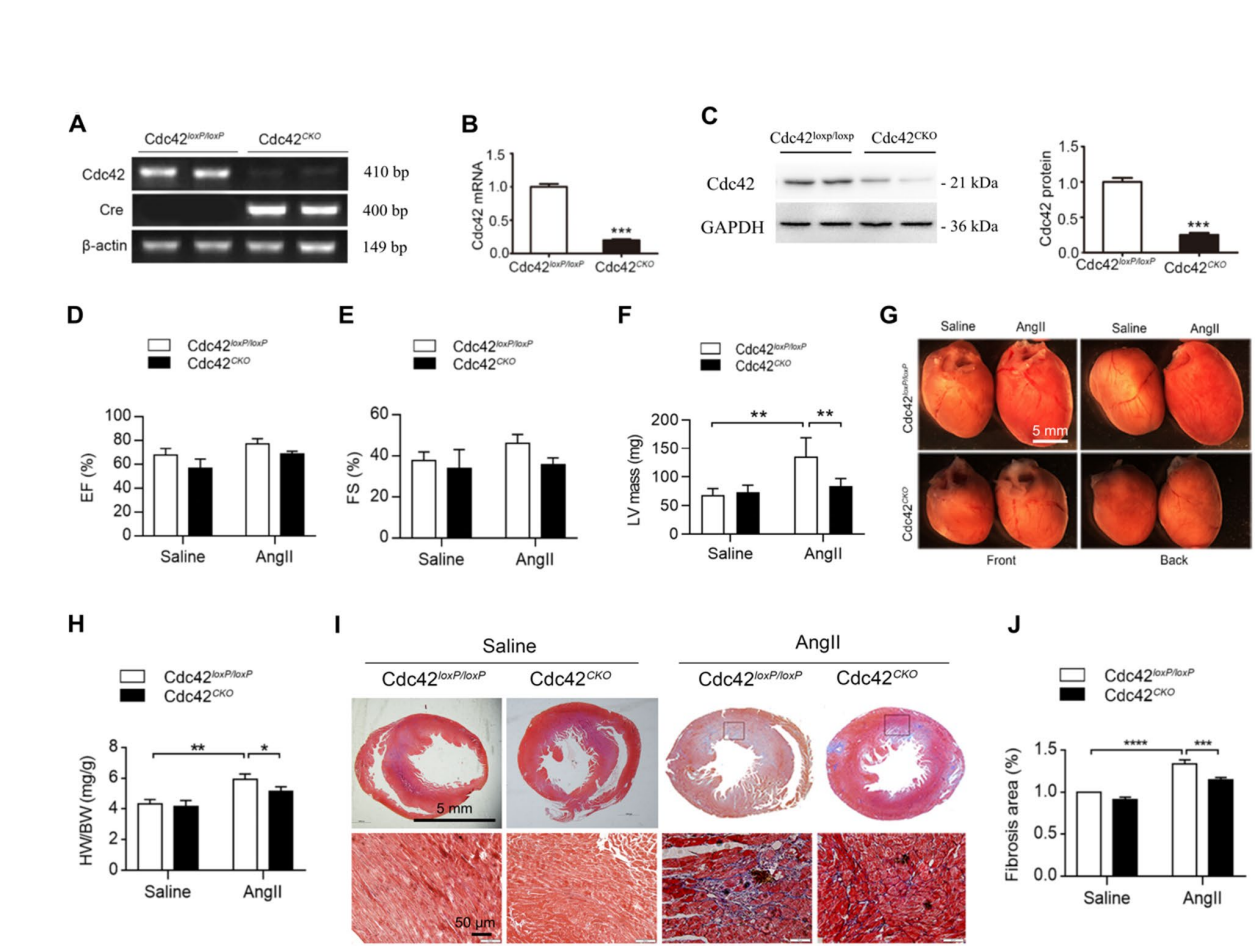
Cardiomyocyte-specific deletion of the Cdc42 gene inhibits AngII-induced cardiac hypertrophy in mice. Cardiomyocyte-specific Cdc42 knockout (Cdc42^*CKO*^) mice were generated by crossing Cdc42^*loxP/loxP*^ mice with MLC2v-Cre mice. The expression of cardiac Cdc42 in heart tissues (C) was measured by semiquantitative qPCR (**A**), RT‒PCR (**B**), or western blotting. The data were analyzed with a t test to evaluate the statistical significance of differences between two groups. The left ventricle (LV) mass (mg), ejection fraction (EF) (%) and fraction shortening (FS) (%) were measured by echocardiography in Cdc42^*loxP/loxP*^ and Cdc42^*CKO*^ mice treated with or without AngII infusion (1500 ng/kg/min) for 7 days in three independent experiments (*n* = 4–6 per group per group, total *n* = 14–15 per group) (**D-F**). Representative images of mouse hearts (**G**), the ratio of heart weight (HW)/body weight (BW) (%) (**H**), and histological images of cardiac fibrosis by Masson’s trichome staining (**I**) and the relative fibrotic area comparison (**J**) are presented for Cdc42^*loxP/loxP*^ and Cdc42^*CKO*^ mice with or without AngII infusion for 7 days. The scale bars are indicated in the figures. Two-way ANOVA and an unpaired two-tailed Tukey test were used to evaluate the statistical significance of differences between the groups. The data are presented as the means ± SDs and were generated from three independent experiments. * *P* < 0.05, **P < 0.01, ***P < 0.001

To determine the role of Cdc42 in the heart, we generated cardiomyocyte-specific Cdc42 knockout (Cdc42^*CKO*^) mice by crossing Cdc42^*loxP/loxP*^ mice with cardiac-specific myosin light chain driven Cre (MLC2v-Cre) mice. Cdc42^CKO^ mice were born with a Mendelian ratio and normal cardiac function. As shown in Fig. 1A, the corresponding cDNA bands for Cdc42 and Cre were identified by RT-PCR in mouse hearts. The protein and mRNA expression levels of cardiac Cdc42 were decreased by 75% and 80%, respectively, in Cdc42^*CKO*^ mice compared with those in Cdc42^*loxP/loxP*^ mice (Fig. 1B and C). The expressions of another two small G proteins in Rho family, RhoA, and Rac1, were also examined and compared. There were no significant differences in the expression of the RhoA protein (Supplementary Figure S1A) or Rac1 mRNA (Supplementary Figure S1B) in the heart tissues between the Cdc42^*CKO*^ and Cdc42^*loxP/loxP*^ mice. These data indicated that cardiomyocyte-specific deletion of the Cdc42 gene did not affect the basal function of the heart in mice.

To determine the effects of Cdc42 on cardiac hypertrophy, Cdc42^*CKO*^ and Cdc42^*loxP/loxP*^ mice were infused with 1500 ng/kg/min AngII for 7 days. Echocardiographic measurements revealed that cardiomyocyte-specific deletion of the Cdc42 gene significantly alleviated AngII-induced increases in left ventricle (LV) mass (Fig. 1F), left ventricle posterior wall thickness at diastole (LVPWthD) and left ventricle posterior wall thickness at systole (LVPWthS) in Cdc42^*CKO*^ mice compared with those in Cdc42^*loxP/loxP*^ mice, whereas other echocardiographic parameters, such as ventricular ejection fraction (EF, Fig. 1D) and fractional shortening (FS, Fig. 1E), were within the normal range (Supplementary Table S1 and Figure S2). In addition, cardiomyocyte Cdc42 deficiency significantly reduced AngII-induced cardiac hypertrophy and decreased heart weight (HW) and body weight (BW) (Fig. 1G and H), although there were no significant differences in AngII-induced increases in systolic/diastolic pressure or heart rate (Supplementary Figure S2B-D) between Cdc42^*CKO*^ mice and Cdc42^*loxP/loxP*^ mice. Moreover, AngII infusion-induced cardiac fibrosis was ameliorated in Cdc42^*CKO*^ mice compared with Cdc42^*loxP/loxP*^ mice (Fig. 1I and J). Thus, these results demonstrated that cardiomyocyte-specific deletion of the Cdc42 gene significantly alleviated AngII-induced cardiac hypertrophy and cardiac fibrosis.

### Cardiomyocyte-specific deletion of Cdc42 specifically inhibited AngII-induced activation of the MEK3/6-p38 signaling pathway in mouse hearts

**Fig. 2:**
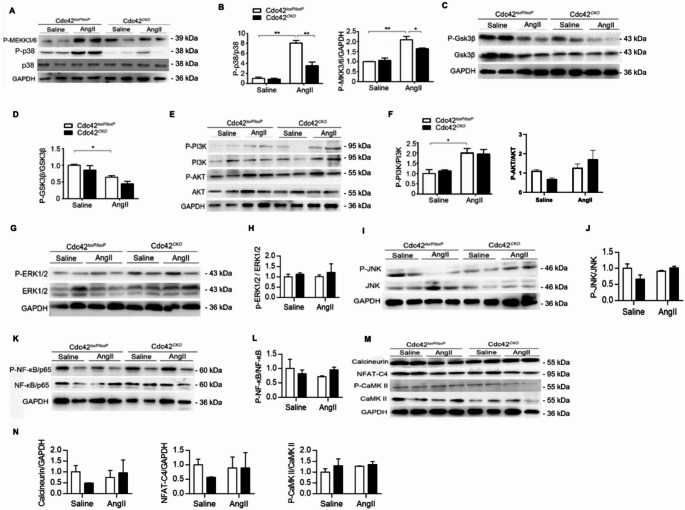
Cardiomyocyte-specific deletion of the Cdc42 gene inhibits AngII-induced activation of the MEKK3/6-p38 signaling pathway in mouse heart tissues. Phosphorylated MKK3/6 and p38 (**A**,** B**), GSK3β (**C**, **D**), PI3K and AKT (**E**, **F**), ERK1/2 (**G**,** H**), JNK (**I**,** J**), NF-κB/P65 (**K**,** L**), calcineurin, NFAT-C4 and CaMK II (**M**,** N**) were quantified by western blot analysis. GAPDH served as a control. Two-way ANOVA and an unpaired two-tailed Tukey test were used to evaluate the statistical significance of differences between the groups. The data are representative of three independent experiments. *P < 0.05, **P < 0.01

Next, we examined hypertrophy-related signaling pathways, including the MAPK and PI3K/Akt pathways, in mouse hearts. The results showed that AngII induced the phosphorylation of MEK3/6 kinase and p38 kinases in hypertrophic mouse hearts, and cardiac Cdc42 deficiency significantly inhibited MEK3/6-p38 activation compared with that in Cdc42^*loxP/loxP*^ hearts (Fig. 2A and B), although there were no alterations in the total protein expression of p38 between Cdc42^*loxP/loxP*^ and Cdc42^*CKO*^ mice after AngII infusion. In addition, AngII significantly inhibited the phosphorylation of GSK3β or increased the phosphorylation of AKT and PI3K in hypertrophic hearts, but there were no significant differences in the total protein expression or phosphorylation of GSK3β, PI3K or AKT in the heart between Cdc42^*loxP/loxP*^ and Cdc42^*CKO*^ mice (Fig. 2C-F). Furthermore, there were no significant differences in ERK1/2, JNK, NF-kB/p65, CaMK II, calcineurin or NFAT-C4 signaling in the heart between Cdc42^*CKO*^ and Cdc42^*loxP/loxP*^ mice after AngII stimulation (Fig. 2G-N). Thus, our results suggested that cardiac deletion of the Cdc42 gene significantly alleviated Ang II-induced cardiac hypertrophy by suppressing the activation of the MKK3/6-p38 signaling pathway in the heart.

In addition, the transcriptomes of mouse heart tissues from the four groups (Cdc42^*loxP/loxP*^ and Cdc42^*CKO*^ after saline infusion and Cdc42^*loxP/loxP*^ and Cdc42^*CKO*^ after AngII infusion) were compared and analyzed. A total of 1588 differentially expressed genes were identified by RNA-Seq analysis between the hearts of Cdc42^*loxp/loxp*^ and Cdc42^*CKO*^ mice subjected to AngII stimulation. The genes related to the differential expression of Cdc42^*loxP/loxP*^ and Cdc42^*CKO*^ among the AngII infusion groups are listed in supplementary table S2, in which the most differentially expressed genes were related to PATH: 04151 (PI3K-Akt signaling pathway), PATH: 04062 (chemokine signaling pathway), and PATH: 04010 (MAPK signaling pathway) in heart tissues according to their gene ontology (GO) categories and KEGG pathways, suggesting that these pathways might be involved in attenuating AngII-induced cardiac hypertrophy due to cardiac Cdc42 deficiency (http://www.genome.jp/kegg/) (Supplementary Figure S3).

### AngII-induced activation of the MEKK3/6-p38 pathway was dependent on Cdc42 in adult cardiomyocytes in vitro

**Fig. 3:**
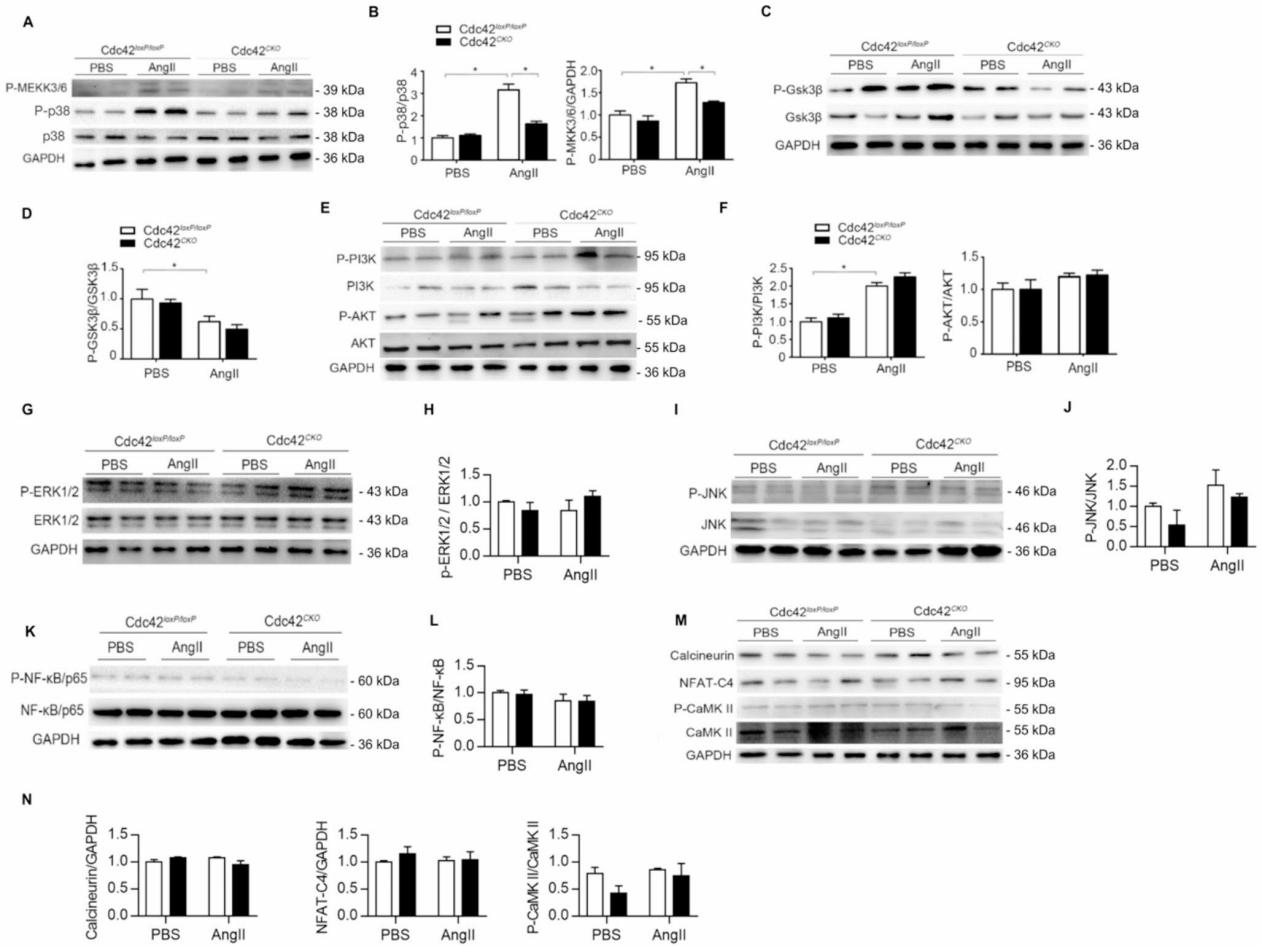
Cdc42 deficiency inhibits AngII-induced activation of the MEKK3/6-p38 signaling pathway in cardiomyocytes. Cardiomyocytes were isolated from adult Cdc42^*LoxP/LoxP*^ and Cdc42^*CKO*^ mice and treated with or without 200 ng/ml AngII for 15 min. Phosphorylated protein levels of MKK3/6 and p38 (**A**,** B**), GSK3β (**C**, **D**), PI3K and AKT (**E**, **F**), ERK1/2 (**G**,** H**), JNK (**I**,** J**), NF-κB/P65 (K, L), calcineurin, NFAT-C4 and CaMK II (**M**,** N**) were quantified by western blot analysis. GAPDH served as a control. Two-way ANOVA and an unpaired two-tailed Tukey test were used to evaluate the statistical significance of differences between the groups. The data are representative of three independent experiments. *P < 0.05

To further confirm the role of Cdc42 in AngII-induced cardiac hypertrophy in vitro, we isolated adult cardiomyocytes from 8 ~ 10-week-old male Cdc42^*loxP/loxP*^ and Cdc42^*CKO*^ mice. Cardiomyocytes were cultured in serum-free medium and harvested for western blot analysis after 15 min of AngII (1000 ng/ml) stimulation. Consistent with the results from the in vivo experiments, Cdc42 deficiency significantly inhibited the AngII-induced phosphorylation of the MEKK3/6 and p38 proteins in cardiomyocytes (Fig. 3A and B). In addition, Cdc42 deficiency did not affect the AngII-induced downregulation of GSK3β phosphorylation (Fig. 3C and D) or the upregulation of PI3K and AKT phosphorylation (Fig. 3E and F) in cardiomyocytes or the total p38, GSK3β, AKT and PI3K protein expression between Cdc42^*loxP/loxP*^ and Cdc42^*CKO*^ cardiomyocytes after AngII stimulation (Fig. 3C-F). Furthermore, there were no significant differences in the phosphorylation or total protein expression of ERK, JNK, NF-κB/p65, CaMKII, NFAT-C4 or calcineurin between Cdc42^*CKO*^ and Cdc42^*loxP/loxP*^ cardiomyocytes with or without AngII stimulation (Fig. 3G-N). These results further confirmed that Cdc42 deficiency inhibited AngII-induced cardiac hypertrophy by selectively inhibiting the MEKK3/6-p38 signaling pathway in vitro.

### Inhibition of Cdc42 or p38 attenuated both Cdc42 overexpression- and AngII-induced cardiomyocyte hypertrophy

**Fig. 4:**
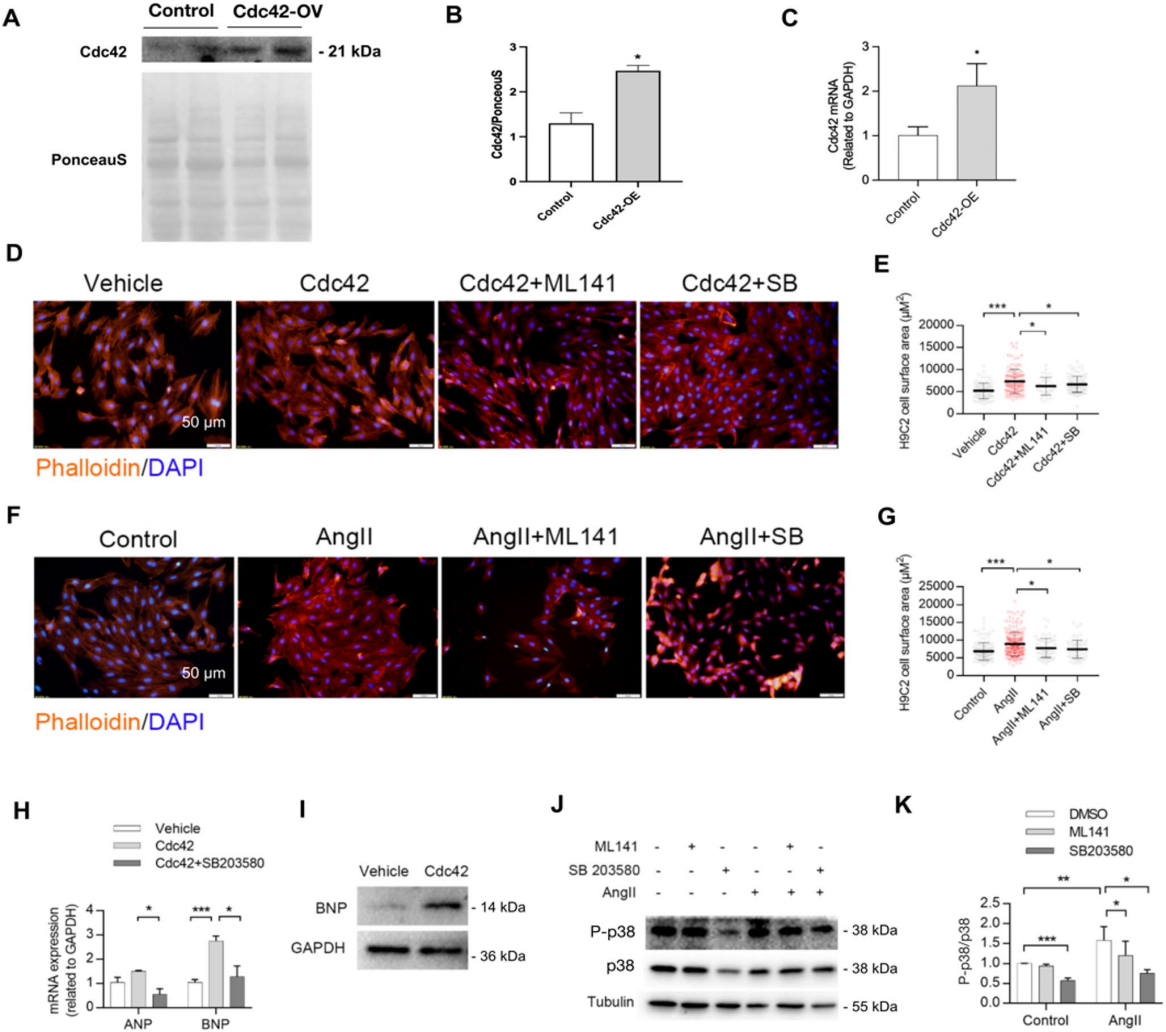
The overexpression of Cdc42 promoted cardiomyocytic hypertrophy by activating the p38 signaling pathway in vitro. H9c2 cells were transfected with the PCMV-Cdc42-myc vector (Cdc42-OV group), stimulated with AngII (200 nmol/L, AngII group) for 24 h, or pretreated with 2 µM ML141 (Cdc42 inhibitor) or 1 µM SB 203580 (p38 inhibitor) for 30 min in a 5% CO_2_ incubator at 37 °C. The cells were subsequently transfected with the PCMV-myc vector as a control (vehicle group). The efficiency of Cdc42 overexpression was confirmed by Western Blot and real-time PCR in H9c2 cell lines transfected with expressing vector of Cdc42 (A-C). Representative immunofluorescence images (red, phalloidine; blue, DAPI) of H9c2 cells with overexpression of Cdc42 (**D**) or AngII stimulation (**F**) and with or without pretreatment with a p38 inhibitor or a Cdc42 inhibitor (**D**,** F**) were acquired, and the cellular surface area was quantified (**E**,** G**). The mRNA (**H**) and protein (**I**) expression levels of reprogrammed hypertrophic genes (ANP and BNP) were quantified by RT‒PCR in Cdc42-OV cells treated with or without SB203580, and GAPDH was used as a reference gene. The total or phosphorylated p38 in H9c2 cells treated with or without ML141 or SB203580 was measured after AngII stimulation, and tubulin was used as a control (**J**, **K**). Two-way ANOVA and an unpaired two-tailed Tukey test were used to evaluate the statistical significance of differences between the groups. The data are representative of three independent experiments. *P < 0.05, **P < 0.01, ***P < 0.001

To further clarify the role of Cdc42 in cardiomyocytes, H9c2 cell lines overexpressing Cdc42 were constructed by transfection with the PCMV-Cdc42-myc vector or the PCMV-myc vector (as a control). The overexpression of cardiomyocytic Cdc42 was confirmed by qPCR and Western blot (Fig. 4A-C). As shown in Fig. 4D and E, immunofluorescence analysis revealed that the overexpression of Cdc42 significantly increased the surface area of H9c2 cells, while ML141 (a Cdc42 inhibitor) and SB 203580 (a p38 inhibitor) strongly inhibited the Cdc42 overexpression-induced increase in cardiomyocytes. In addition, ML141 and SB 203580 remarkably inhibited the AngII-induced increase in H9c2 cells (Fig. 4F and G). Furthermore, western blot and qPCR analyses showed that the overexpression of Cdc42 increased the expression of hypertrophic genes such as ANP and BNP, which were significantly suppressed by SB203580 (Fig. 4H and I), whereas ML141 and SB203580 markedly inhibited the AngII-induced phosphorylation of p38 in H9c2 cells (Fig. 4J and K). These results further demonstrated that Cdc42 overexpression- or AngII-induced cardiomyocyte hypertrophy was dependent on activation of the p38 signaling pathway.

### Cardiac-specific deletion of Cdc42 protected cardiac function from TAC-induced cardiac remodeling in mice

**Fig. 5:**
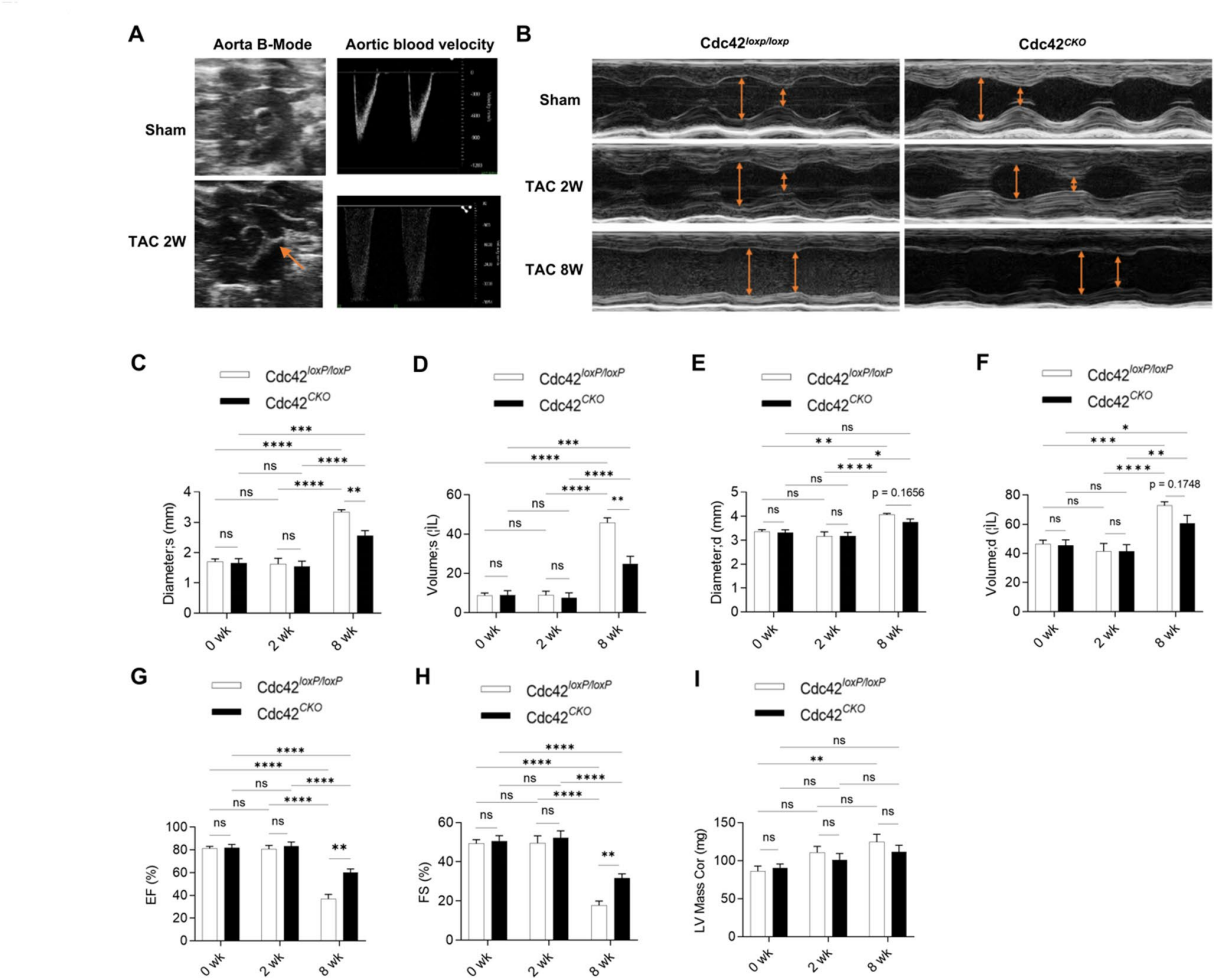
Cardiac-specific deletion of Cdc42 protects cardiac functions from TAC-induced cardiac remodeling. Representative echocardiography images of a restricted mouse aorta (as indicated by the orange arrow) and increased aortic blood flow are presented as indicators of successful TAC modeling in mice (**A**). Echocardiograms were obtained at 2 or 8 weeks after TAC in Cdc42^*loxP/loxP*^ and Cdc42^*CKO*^ mice (*n* = 6–8 per group); representative images are shown (**B**). The systolic diameter (diameter; s) and volume (volume; s), diastolic diameter (diameter; d) and volume (volume; d), ejection fraction (EF) (%), fraction shortening (FS) (%) and LV mass were measured by echocardiography in Cdc42^*loxP/loxP*^ and Cdc42^*CKO*^ mice (**C-I**). Two-way ANOVA and an unpaired two-tailed Tukey test were used to evaluate the statistical significance of differences between the groups. *P < 0.05, **P < 0.01, ***P < 0.001

**Fig. 6:**
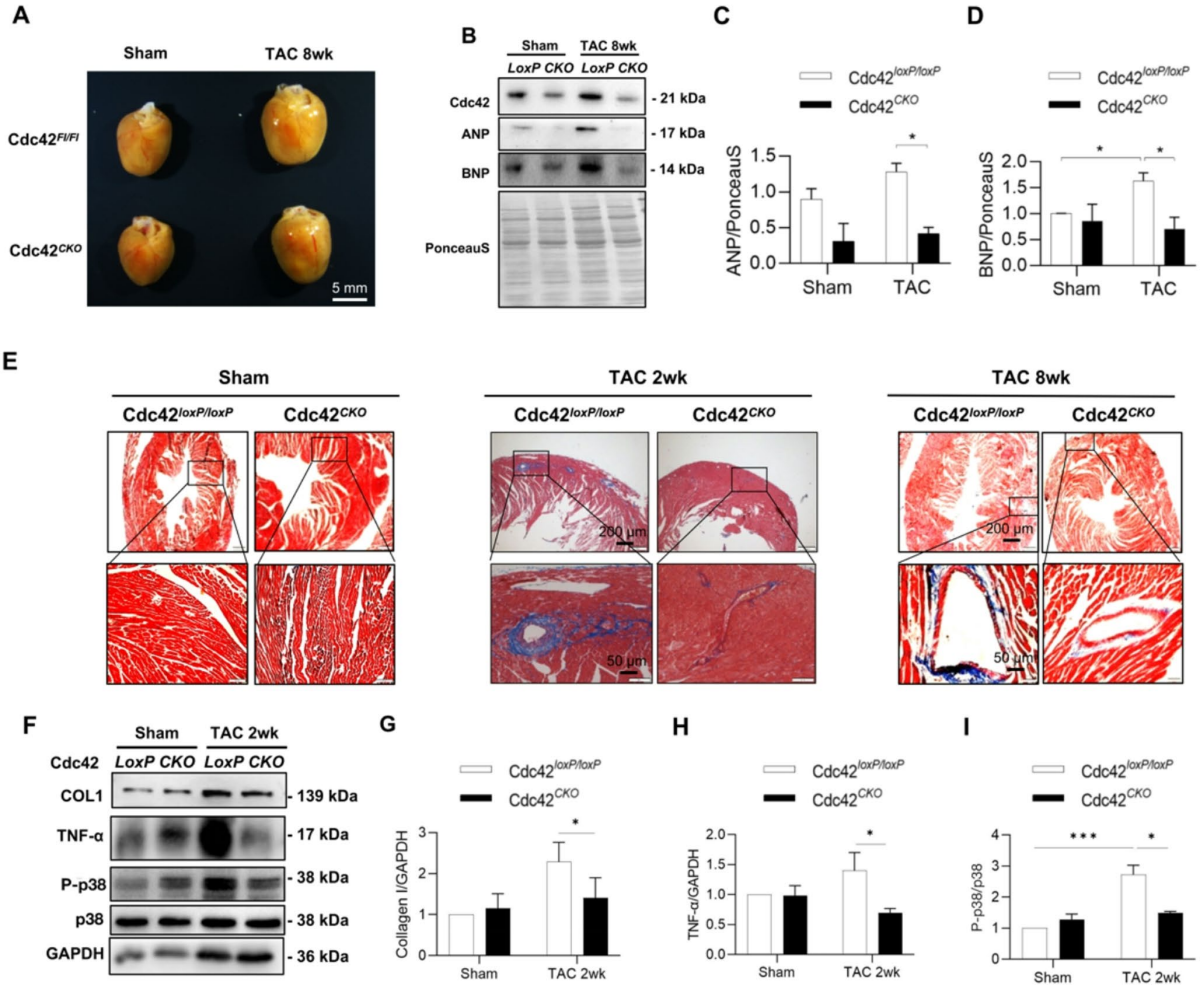
Cardiac Cdc42 deficiency inhibited cardiac hypertrophy and fibrosis and p38 phosphorylation in TAC-induced cardiac remodeling. Representative images of mouse hearts were obtained at 8 weeks after TAC (**A**). The protein expression levels of Cdc42, ANP and BNP were quantitatively analyzed in TAC-8wk hearts (**B-D**). Histological images of cardiac fibrosis in Cdc42 loxP/loxP and Cdc42 CKO mice are shown in blue by Masson’s trichrome staining (**E**). The protein expression levels of Collagen I, TNF-α, p38 and p-p38 were quantitatively analyzed in heart tissues from TAC-2-week-old mice (**F-I**)**.** The data are representative of three independent experiments. *P < 0.05, **P < 0.01, ***P < 0.001

Several groups have determined that Cdc42 might promote hypertrophy in cardiomyocytes [13], whereas Maillet M et al. reported that heart-specific deletion of Cdc42 aggravated TAC-induced cardiac hypertrophy and promoted the transition to heart failure in Cdc42^αMHC−Cre^ mice [15]. To further evaluate the role of Cdc42 in cardiac remodeling and cardiac function, a classic model of cardiac remodeling in Cdc42^*CKO*^ mice was generated by transverse aortic constriction for 2 or 8 weeks (Fig. 5A and B). Cardiomyocyte-specific deletion of the Cdc42 gene markedly alleviated TAC-induced dilation of the left ventricle (Fig. 5C-F), improved the cardiac ejection fraction (EF) and fractional shortening (FS) (Fig. 5G and H), and slightly reduced the TAC-induced increase in the LV mass (111.46+/−9.00 versus 124.84+/−9.98, in mg) after TAC for 8 weeks (Fig. 5I and Supplementary Figure S4A). Furthermore, our results showed that cardiac Cdc42 deficiency ameliorated TAC-induced cardiac hypertrophy and inhibited TAC-induced increases in the expression of hypertrophic genes such as ANP and BNP (Fig. 6A-D). In addition, Cdc42 deficiency inhibited TAC-induced cardiac fibrosis in mice (Fig. 6E). However, there was significantly increased cardiac MDA content, but not different between Cdc42^loxP/loxP^ and Cdc42^CKO^ groups post TAC 2wk modeling (Supplementary Figure S4B). Moreover, our results also showed that cardiac Cdc42 deficiency significantly inhibited cardiac collagen I expression and p38 phosphorylation in a mouse model of transverse aortic constriction (TAC) (Fig. 6F-I). These results indicated that cardiac deletion of Cdc42 inhibited TAC-induced cardiac hypertrophy and fibrosis.

### Cardiac Cdc42 deficiency suppressed phospho-p38 and apoptosis in TAC myocardium and reduced the secretion of proinflammatory cytokines in cardiac remodeling

**Fig. 7:**
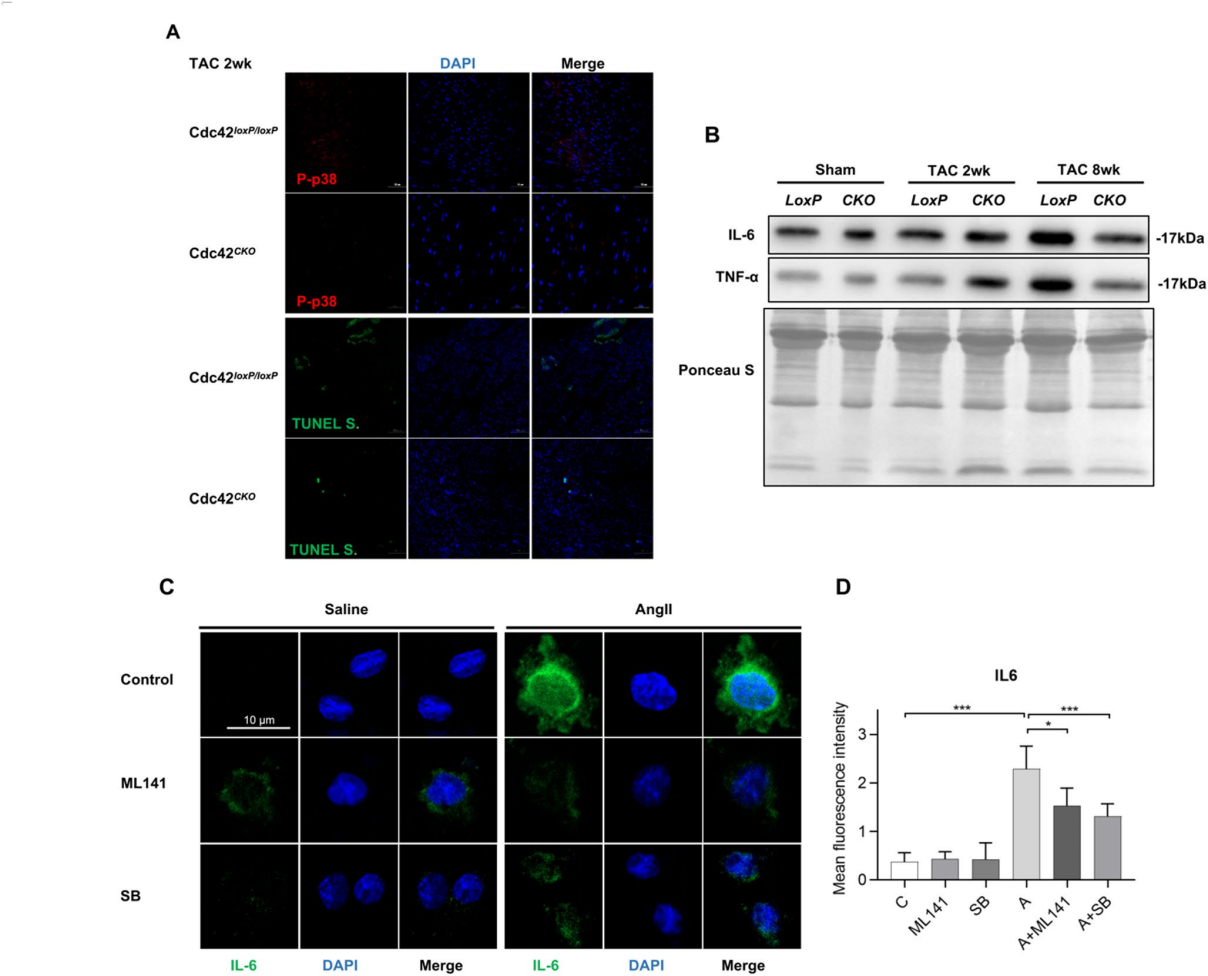
Cardiac Cdc42 deficiency suppresses phospho-p38 and apoptosis in TAC myocardium and reduces the secretion of the proinflammatory cytokines IL6 and TNFα in cardiac remodeling. The representative images of phospho-p38 and TUNEL signals in TAC 2 wk hearts (**A**). The serum levels of IL-6 and TNF-α were quantitatively analyzed at 2 and 8 weeks after TAC (**B**)**.** H9c2 cells were plated on glass coverslips and stimulated with 200 nM AngII for 12 h with or without pretreatment with ML141 (Cdc42 inhibitor) or SB203580 (p38 inhibitor) for 30 min. IL6 expression or quantification was performed by immunofluorescence staining (**C-D**), and the immunofluorescence intensity was recorded and analyzed by ImageJ software (**D**). The data are representative of three independent experiments. *P < 0.05, **P < 0.01, ***P < 0.001

It has been reported that p38 activation has a apoptotic and profibrotic effect by inducing apoptosis and the release of TNF-α and IL-6 in cardiomyocytes, which are closely associated with the development of fibrosis, adverse cardiac remodeling, and heart failure [25]. To further understand whether the role of Cdc42 in cardiac remodeling involves apoptosis and proinflammatory cytokines via p38 signaling, we performed apoptosis assay in TAC 2 wk hearts and examined the levels of proinflammatory cytokines in mouse serum after TAC for 2 or 8 weeks. The results showed that phosphor-p38 and apoptosis were suppressed in TAC 2 wk Cdc42^*CKO*^ hearts, and the serum IL6 and TNFα levels were decreased in TAC 8 wk Cdc42^*CKO*^ mice (Fig. 7A and B). Furthermore, both M141 (a Cdc42 inhibitor) and SB203580 (a p38 inhibitor) markedly reduced the release of IL-6 in H9c2 cells after AngII stimulation (Fig. 7C and D). Thus, these results suggested that cardiac Cdc42 deficiency suppressed apoptosis and the secretions of proinflammatory cytokines IL6 and TNFα via inhibiting the p38 signaling pathway in cardiac remodeling.

## Discussion

Cardiovascular diseases are the most threatening diseases in human health, among which heart failure is the main cause of high mortality. Many heart diseases can lead to heart failure with myocardial hypertrophy and remodeling [26]. Pathological cardiac hypertrophy is usually triggered by mechanical pressure overload or neuroendocrine hormones such as Ang II, endothelin 1, and catecholamines [1]. Transverse aortic constriction (TAC) or angiotensin II infusion, which are involved in mechanical and hormonal signals, respectively, are commonly used to induce cardiac hypertrophy and remodeling in mouse models. Cdc42, a small GTPase, belongs to the Rho family and functions as a molecular switch in cell cycle division, cytoskeleton arrangement, cell polarization, membrane trafficking and signal transduction [5]. In the present study, we investigated the role of Cdc42 in the development of cardiac hypertrophy and fibrosis with TAC- and AngII-induced cardiac remodeling in mice and further explored the relative mechanisms in cardiomyocytes under AngII-treatment in vitro. We demonstrated that cardiac deficiency of Cdc42 significantly prevented both TAC or AngII mediated hypertrophy response and fibrosis and improved cardiac function by blocking the MKK3/6-p38 cascade in cardiac remodeling mice. We also found that Cdc42-mediated MKK3/6-p38 cascade was critical for AngII-induced cardiac hypertrophy in vitro. Furthermore, overexpression of Cdc42 or AngII administration promoted hypertrophy of cardiomyocytes through activating MKK3/6-p38 pathway in H9c2 cells, whereas inhibition of Cdc42 or p38 significantly alleviated overexpression of Cdc42 or AngII-induced hypertrophy in cardiomyocytes.

As a member of the Rho family, Cdc42 is critical for embryonic cardiomyocyte proliferation and heart development [11]. However, the role of Cdc42 in pathological cardiac hypertrophy and cardiac remodeling has been reported to be contradictory. Constitutively activated Cdc42 enhances cardiomyocyte hypertrophy, and downregulated or inhibited Cdc42 activity attenuates LIF-induced cardiomyocyte elongation or high salt-induced cardiac hypertrophy and fibrosis [13, 27]. Conversely, Maillet et al. observed the protective role of Cdc42 in cardiac hypertrophy via JNK activation in heart tissues in a TAC model since TAC-induced cardiac hypertrophy was exacerbated in mice with cardiac-specific Cdc42 deletion, which strongly expresses αMHC-Cre [15]. In our present study, compared with Cdc42 ^loxp/loxp^ mice, Cdc42^*CKO*^ mice with cardiac MLC2v Cre exhibited normal development, breeding behaviors and cardiac functions under unstimulated conditions. Notably, the deletion of Cdc42 significantly decreased TAC- and AngII-induced cardiac hypertrophy and remodeling, as indicated by decreases in the LV mass, the HW/BW ratio and fibrosis in the hearts of Cdc42^*CKO*^ mice. Our results are in agreement with those of the study by Birnbaum group, in which Cdc42 expression was greatly enhanced and was found to be responsible for cardiac hypertrophy and fibrosis, and HS-induced cardiac hypertrophy was prevented by the Cdc42-specific inhibitor ML141 in rats [27]. Interestingly, in Maillet’s study, activation of Cdc42 (Cdc42-GTP, activated state of Cdc42) was increased in neonatal rat cardiomyocytes, while stimulation with LIF, AngII, PE, or ISO, and Cdc42-GTP was also increased in the hearts of WT mice subjected to TAC compared with those of sham-operated mice [15]. The discrepancy in the role of Cdc42 in cardiac hypertrophy and fibrosis might be attributed to the use of different genetic mouse models and the complicated signaling pathways involved in the role of Cdc42 in cardiac hypertrophy.

Several signaling pathways involved in cardiac hypertrophy include the MAPK signaling, PI3K/AKT signaling, Gsk3β signaling, NF-κB signaling, calcineurin/NFAT signaling and CaMK II signaling pathways [1, 28]. The MAPK signaling pathway consists of a sequence of successively functioning kinases that ultimately result in the dual phosphorylation and activation of ERK, JNK and p38 [29]. Cdc42 can activate both the MAPK subfamily and the ERK cascade through PAK, and cooperation with Raf to activate ERK and Cdc42 has also been implicated in the activation of JNK and p38; however, the underlying mechanism is unclear [8, 30–32]. Our data showed that Cdc42 deletion significantly reduced the phosphorylation of p38 in AngII-induced cardiac hypertrophy but did not affect the ERK or JNK pathways, which is consistent with the findings of Pellieux [29]. Furthermore, our data showed that the overexpression of Cdc42 or the administration of AngII promoted cardiomyocyte hypertrophy and increased the phosphorylation of p38 in H9c2 cells. More importantly, the inhibition of Cdc42 (ML141, a Cdc42 inhibitor) or p38 (SB203580, a p38 inhibitor) significantly alleviated the hypertrophy of cardiomyocytes. These observations are supported by previous studies in which Liao et al. reported that targeted activation of p38 contributes to the cardiac remodeling process in the failing heart by promoting interstitial fibrosis and increasing the expression of hypertrophy genes (atrial natriuretic factor, ANF and bMHC) in vivo [33]. In cardiomyocytes, activation of p38 is required for ANF expression induced by hypertrophic agonists, and JNK exerts the opposite effects on the development of myocyte hypertrophy [34].

Most of the evidence points to a pro-hypertrophy, proinflammatory and pro-fibrotic effect of p38 activation on the onset of HF and arrhythmias, which involves cardiac fibrosis, alterations to Ca^2+^-handling proteins, and the modulation of gap junctions in cardiomyocytes [3, 35]. Our results also demonstrated that Cdc42 deficiency may ameliorate cardiac fibrosis and improve contractile function during mouse heart remodeling. MKK3bE and MMK6bE are upstream kinases of p38 MAPK, and their overexpression significantly increased p38 kinase activity, the expression of ANP/BNP and interstitial fibrosis in the heart [36]. In addition, the activation of p38 via MKK6bE may mediate the inflammatory induction of TNF-α and IL-6 in cardiomyocytes and contribute to the development of fibrosis, adverse cardiac remodeling, and heart failure [25, 36]. A p38 specific inhibitor SB 203580 significantly reduced myocardial apoptosis and improved cardiac function recovery after reperfusion [37]. And specific degradation of phosphor-p38 notably reduced proinflammatory cytokines, IL6, IL-1β and TNFα in diverse brain cells [38]. Our results suggest that cardiac Cdc42 deficiency might reduce hypertrophy response, the apoptosis and the expression and release of proinflammatory cytokines in cardiac remodeling or cardiomyocytes and Cdc42 deficiency or inhibition of Cdc42 activity suppressed MKK3/6-p38 activation both *in vivo and in vitro*. In addition, cardiac Cdc42 deficiency attenuated both AngII and TAC mediated cardiac remodeling. Since TAC modeling with pressure overload (due to blockage of blood flow in aorta) induced overactivation of the neuroendocrine system and lead to elevated circulating levels of Ang II and norepinephrine in vivo [39], the mechanism with AngII activated signaling pathway might be shared in both AngII and TAC modeling. In this study, we found that Cdc42 might serve as an upstream regulator of p38 to modulate MKK3/6-p38 signaling pathway in cardiac remodeling since there were not significantly different in other pathway between the control and Cdc42 deletion group. In addition, our results indicated that other factors such as proinflammatory cytokines might also be involved in the role of Cdc42 in cardiac remodeling. It might be worth investigating in the future.

Taken together, our results demonstrated that the Cdc42-mediated MKK3/6-p38 cascade was critical for cardiac hypertrophy and cardiac fibrosis, and our data emphasized that Cdc42 is a hypertrophic and profibrotic molecule that regulates MKK3/6-p38 signaling in cardiac remodeling.

## Conclusions

In the present study, we demonstrated that cardiac-specific deletion of Cdc42 significantly alleviated cardiac hypertrophy in vivo and in vitro by blocking the MKK3/6-p38 cascade. The overexpression of Cdc42 enhanced the surface area and hypertrophic gene expression in cardiomyocytes by activating p38. More importantly, Cdc42 deficiency inhibited TAC-induced cardiac remodeling and improved cardiac function in mice. Therefore, we concluded that Cdc42 plays a key role in cardiac hypertrophy and remodeling by activating the MKK3/6-p38 signaling pathway, and our study provides insight into the mechanism of cardiac hypertrophy and remodeling.

## Electronic supplementary material

Below is the link to the electronic supplementary material.

Supplementary Material 2

Supplementary Material 3

## Data Availability

The data sets used and/or analyzed during the current study are available from the corresponding authors upon reasonable request.
