# Supplementary material for: Cell division cycle protein 42-driven activation of the MKK3/6-p38 signaling pathway participates in cardiac remodeling in mice
Source: Cell Mol Life Sci. 2025 Jul 3;82(1):269. doi: 10.1007/s00018-025-05743-4 (PMC12229389; doi:10.1007/s00018-025-05743-4)
Supplement: Supplementary file 1 — Supplementary Material 2 [file 18_2025_5743_MOESM1_ESM.docx]

**Supplementary Materials**

**Cell division cycle protein 42-drived activation of MKK3/6-p38 signaling pathway participates in cardiac remodeling in mice**

Ke Wen^a,b,d #^, Lin Xie^a,c,e #^, Quan-Wen Liu^a #^, Guan-Hui Yu^a,b^, Xu-Hui Qiao^a,c^, Yu-Chun Huang^a^, Lu Wang^a^, Xin Li^a,c^, Li-Dan Wen^a^, Xiao-Lei Wang^a^, Jing He^a^, Xin-Yu Xiao^a^, Xiao-Xiao Zhao^a^, Ling-Fang Wang^a^, Hong-Bo Xin^a,b,c,*^ and Ke-Yu Deng^a,b,c,*^

^a^The National Engineering Research Center for Bioengineering Drugs and the Technologies, Institute of Translational Medicine, ^b^School of Pharmacy, ^c^School of Life and Science, Nanchang University, Nanchang 330031, P.R.China, ^d^Jiangxi University of Traditional Chinese Medicine, ^e^Jiangxi Provincial People's Hospital, The First Affiliated Hospital of Nanchang Medical College, Nanchang 330031, P.R.China.

^#^These authors equally contributed to this work.

^*^Correspondence to: Ke-Yu Deng (email: [dky@ncu.edu.cn](mailto:dky@ncu.edu.cn), tel: 86-79183827160) and Hong-Bo Xin (email: [xinhb@ncu.edu.cn](mailto:xinhb@ncu.edu.cn), tel: 86-791-83827168), Institute of Translational Medicine, Nanchang University, No. 1299 Xuefu Road, Honggutan District, Nanchang, Jiangxi Province 330031, P. R. China.


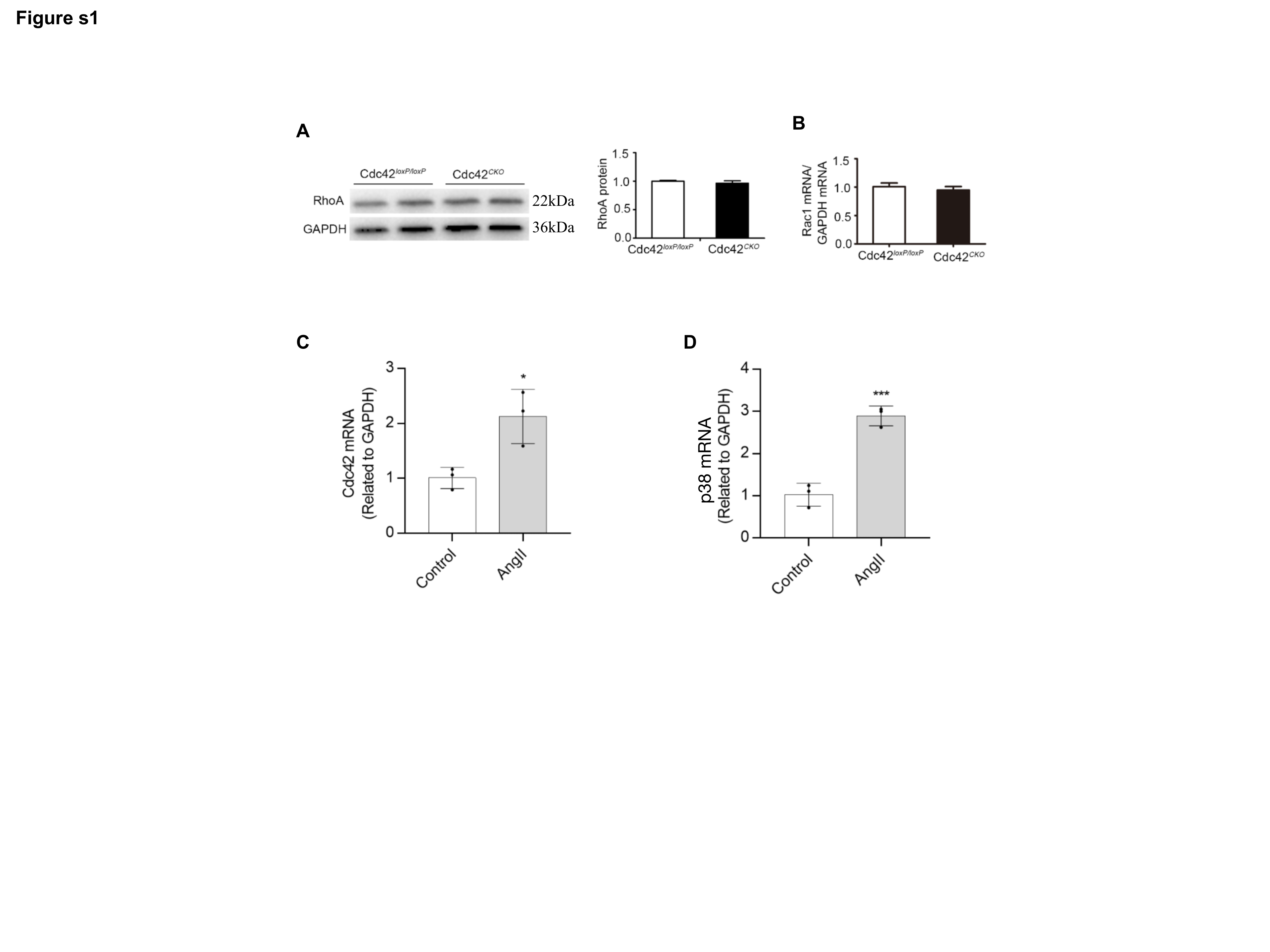


**Figure S1.** The protein expression of RhoA was determined in heart tissues of Cdc42*^loxP/loxP^* and Cdc42*^CKO^* mice, GAPDH as a control (**A**). The mRNA expression of Rac1, Cdc42 and p38 was detected by RT-PCR in heart tissues from Cdc42*^loxP/loxP^* and Cdc42*^CKO^* mice (**B-D)**.

**
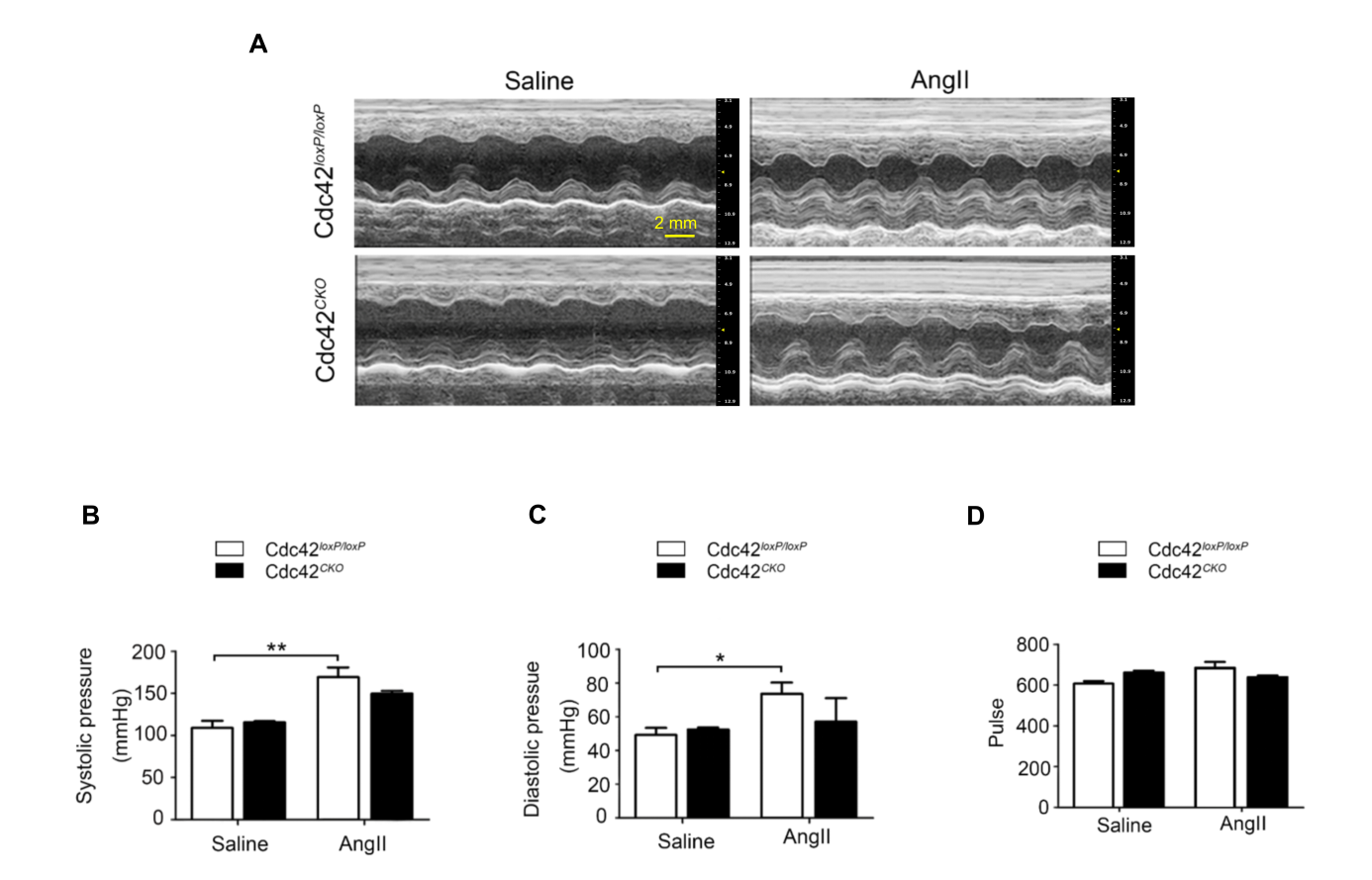
**

**Figure S2.** The representative images of echocardiographic M-model measurement were taken and presented from Cdc42*^loxP/loxP^* and Cdc42*^CKO^* mice with or without AngII infusion for 7 days. (**A)**. The measurements of systolic pressure (**B**), diastolic pressure (**C**) and pulses (**D**) were measured by tail-off method in mice and analyzed (**B-D**).

**
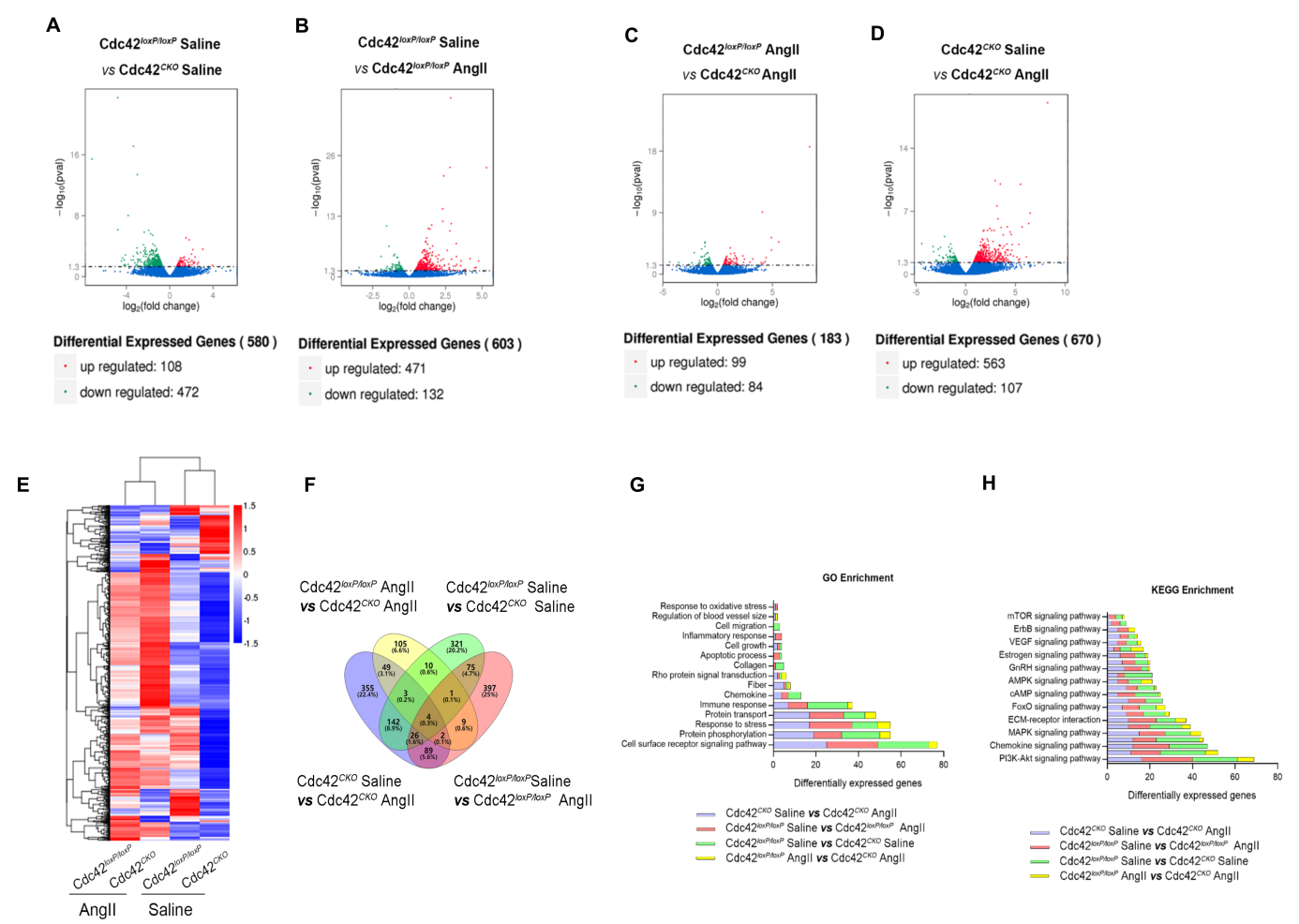
**

**Figure S3. Comparisons and analyses of mouse heart transcriptomes and associated pathways between exprimental groups.** The volcano plots of RNA-Seq represented the comparisons of differential expressed genes in heart tissues between the groups of Cdc42*^loxP/loxP^* and Cdc42*^CKO^* mice with or without AngII infusion for 7 days (upregulated genes in red, and downregulated genes in green) (**A-D**). The heatmap of RNA-Seq from heart tissues of Cdc42*^loxP/loxP^* and Cdc42*^CKO^* mice with or without AngII infusion for 7 days (red, upregulated genes; blue, downregulated genes) (**E**). Venn diagram of overlapped differential expression genes in mouse hearts between experimental groups (**F)**. Analyses of Gene ontology (GO) and KEGG pathways of differential expression genes in experimental groups (**G**, **H**). N=3 per group.

**
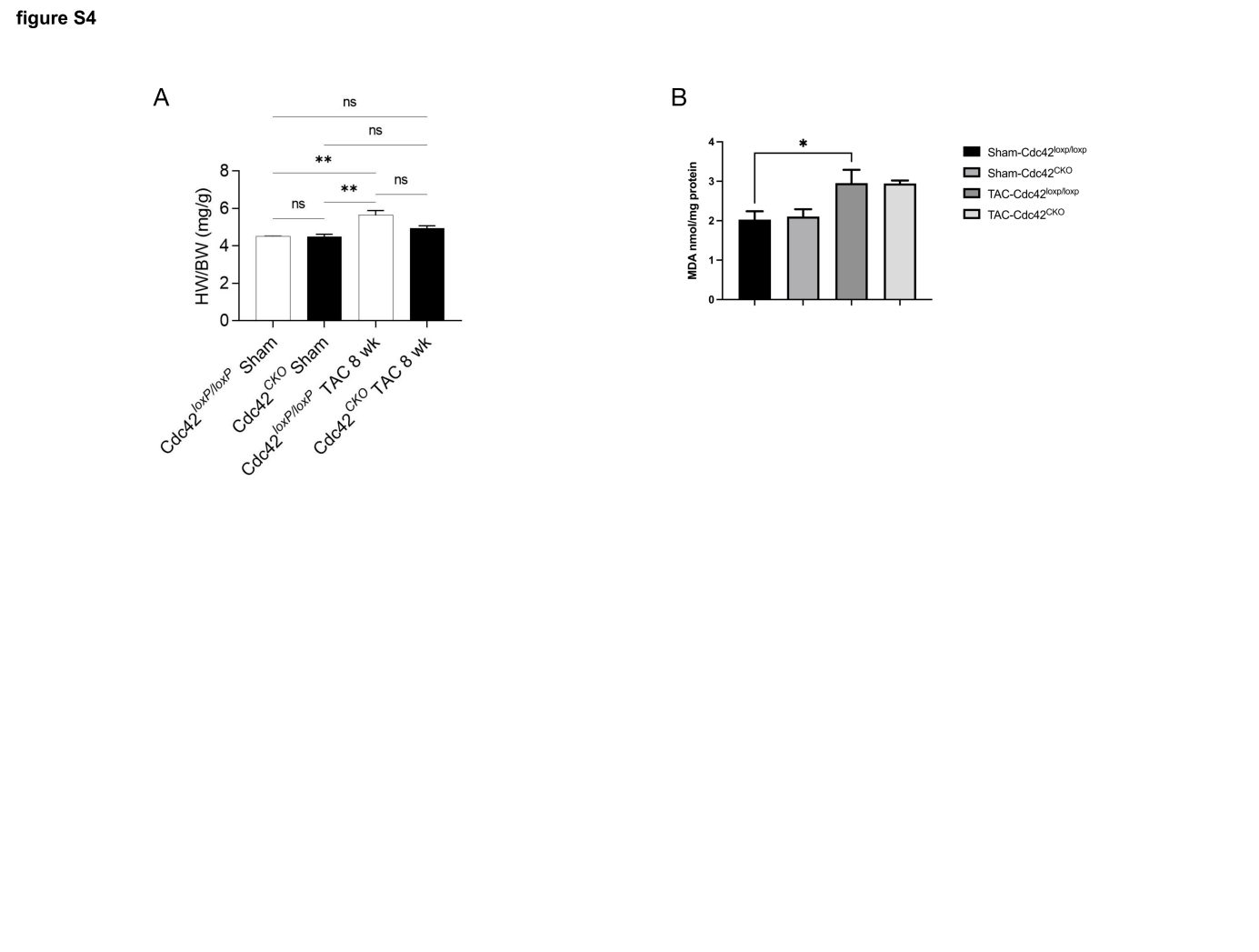
**

**Figure S4. (A)** The ratio of heart weight (HW)/body weight (BW) (%) was measured after TAC 8wk in Cdc42^loxP/loxP^ and Cdc42^CKO^ mice. (**B**) The formation of MDA in the heart tissues of Cdc42^loxP/loxP^ and cdc42^CKO^ mice after TAC 2wk was quantitatively determined and analyzed with two-way ANOVA method. N=3 per group.


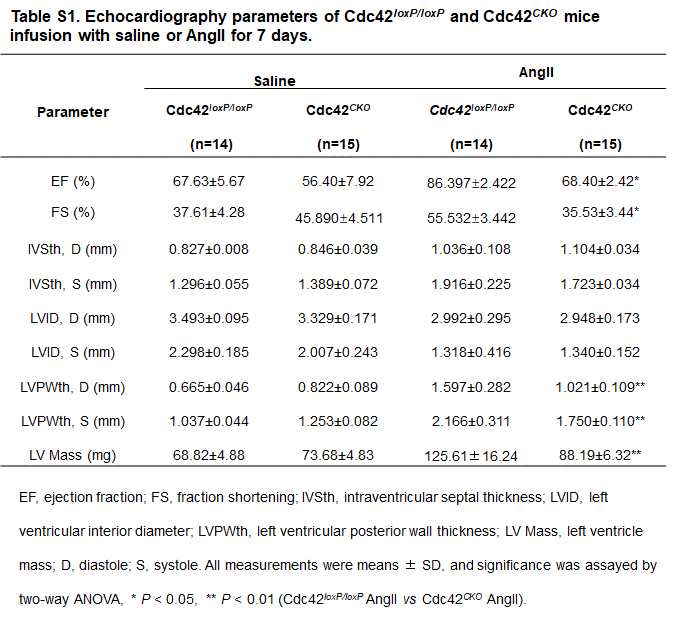


**Table S2. Differential expression genes between Cdc42*^CKO^* VS Cdc42*^loxP/loxP^* heart tissues after AngII infusion for 7 days.**

| **GeneID** | **Gene symbol** | **Description** | **Up-Down** |
| --- | --- | --- | --- |
| ENSMUSG00000035783 | Acta2 | Actin Alpha 2, Smooth Muscle | Up |
| ENSMUSG00000074923 | Pak6 | p21 (RAC1) activated kinase 6 | Up |
| ENSMUSG00000009376 | Met | MET proto-oncogene, receptor tyrosine kinase | Up |
| ENSMUSG00000038793 | Lefty1 | left-right determination factor 1 | Up |
| ENSMUSG00000053716 | Dusp7 | dual specificity phosphatase 7 | Up |
| ENSMUSG00000041417 | Pik3r1 | phosphoinositide-3-kinase regulatory subunit 1 | Up |
| ENSMUSG00000094686 | Ccl21a | C-C motif chemokine ligand 21 | Down |
| ENSMUSG00000029084 | Cd38 | CD38 molecule | Down |
| ENSMUSG00000006699 | Cdc42 | Cell division cycle 42 | Down |
| ENSMUSG00000020108 | Ddit4 | DNA damage inducible transcript 4 | Down |
| ENSMUSG00000031712 | IL15 | interleukin 15 | Down |
| ENSMUSG00000031849 | Comp | cartilage oligomeric matrix protein | Down |
| ENSMUSG00000001494 | Sost | sclerostin | Down |
| ENSMUSG00000007613 | Tgfbr1 | transforming growth factor beta receptor 1 | Down |
| ENSMUSG00000021702 | Thbs4 | thrombospondin 4 | Down |
| ENSMUSG00000027995 | Tlr2 | toll like receptor 2 | Down |
