# Supplementary material for: Cell division cycle protein 42-driven activation of the MKK3/6-p38 signaling pathway participates in cardiac remodeling in mice
Source: Cell Mol Life Sci. 2025 Jul 3;82(1):269. doi: 10.1007/s00018-025-05743-4 (PMC12229389; doi:10.1007/s00018-025-05743-4)

Figure 1

C

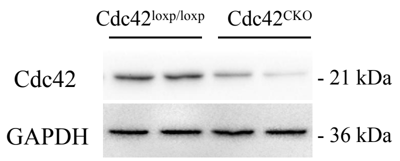

Cdc42
21 kD

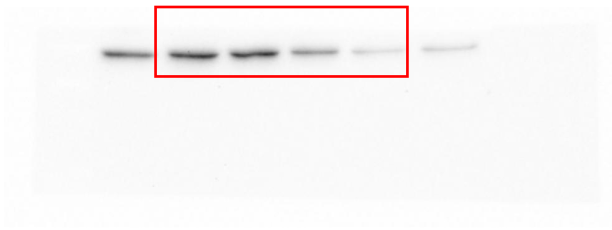

GAPDH
36 kD

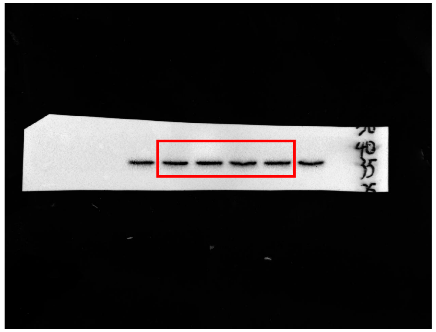

Figure 2

A

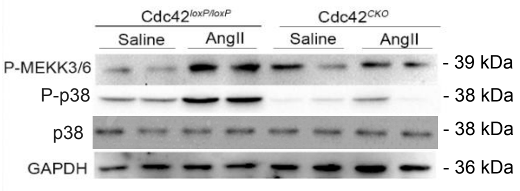

P-MEKK3/6
39 kD

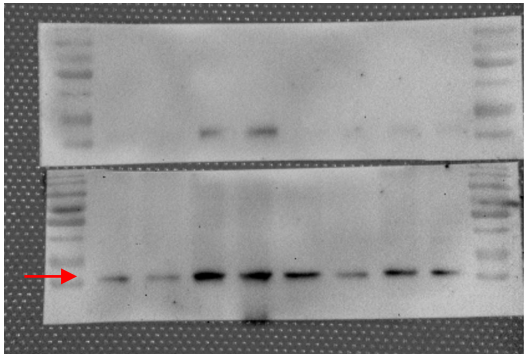

P38
38 kD

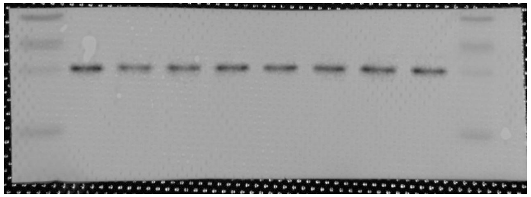

P-P38
38 kD

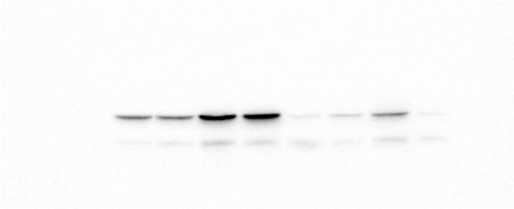

GAPDH
36 kD

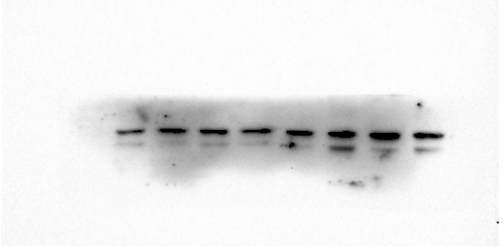

c

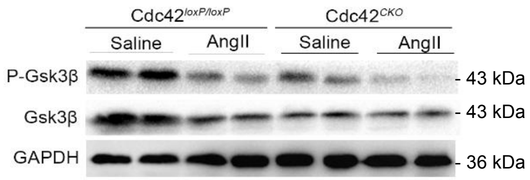

P-Gsk3β
43 kD

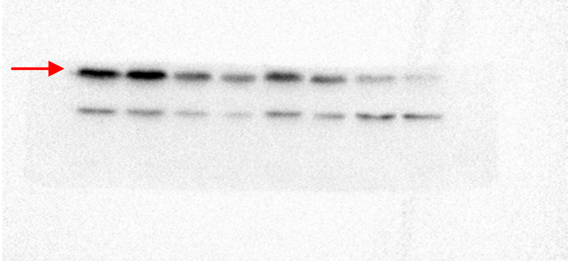

Gsk3β
43 kD

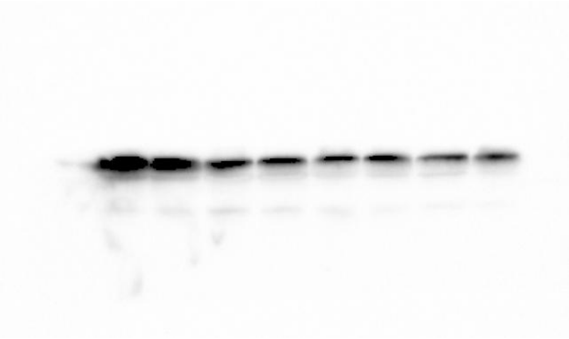

GAPDH
36 kD

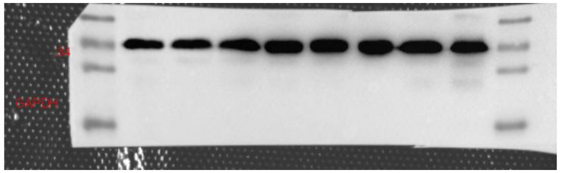

Figure 2

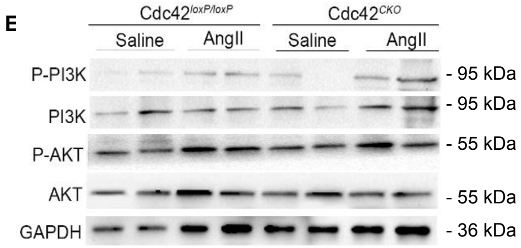

P-PI3K
95 kD

PI3K
95 kD

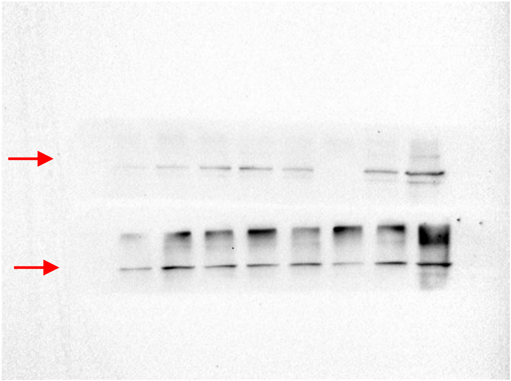

GAPDH
36 kD

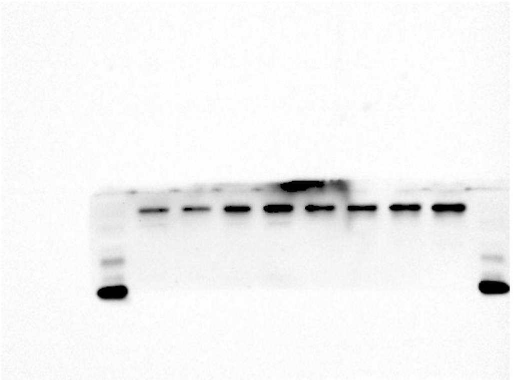

P-AKT
55 kD

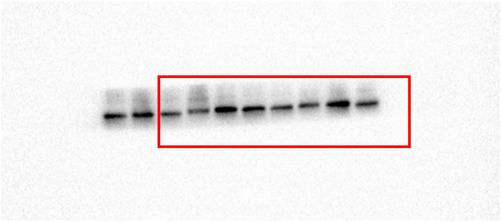

AKT
55 kD

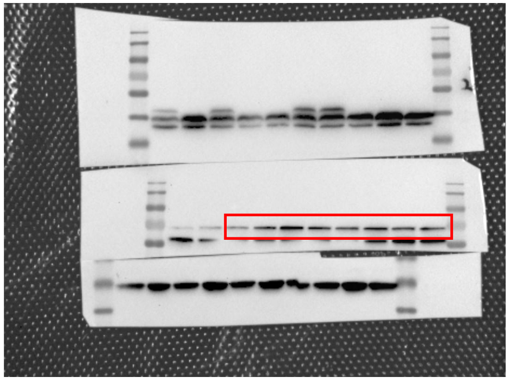

GAPDH
36 kD

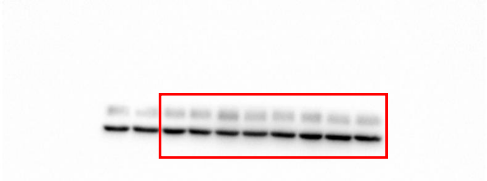

This figure displays p-AKT and AKT loading controls, but was not included in the manuscript.

Figure 2

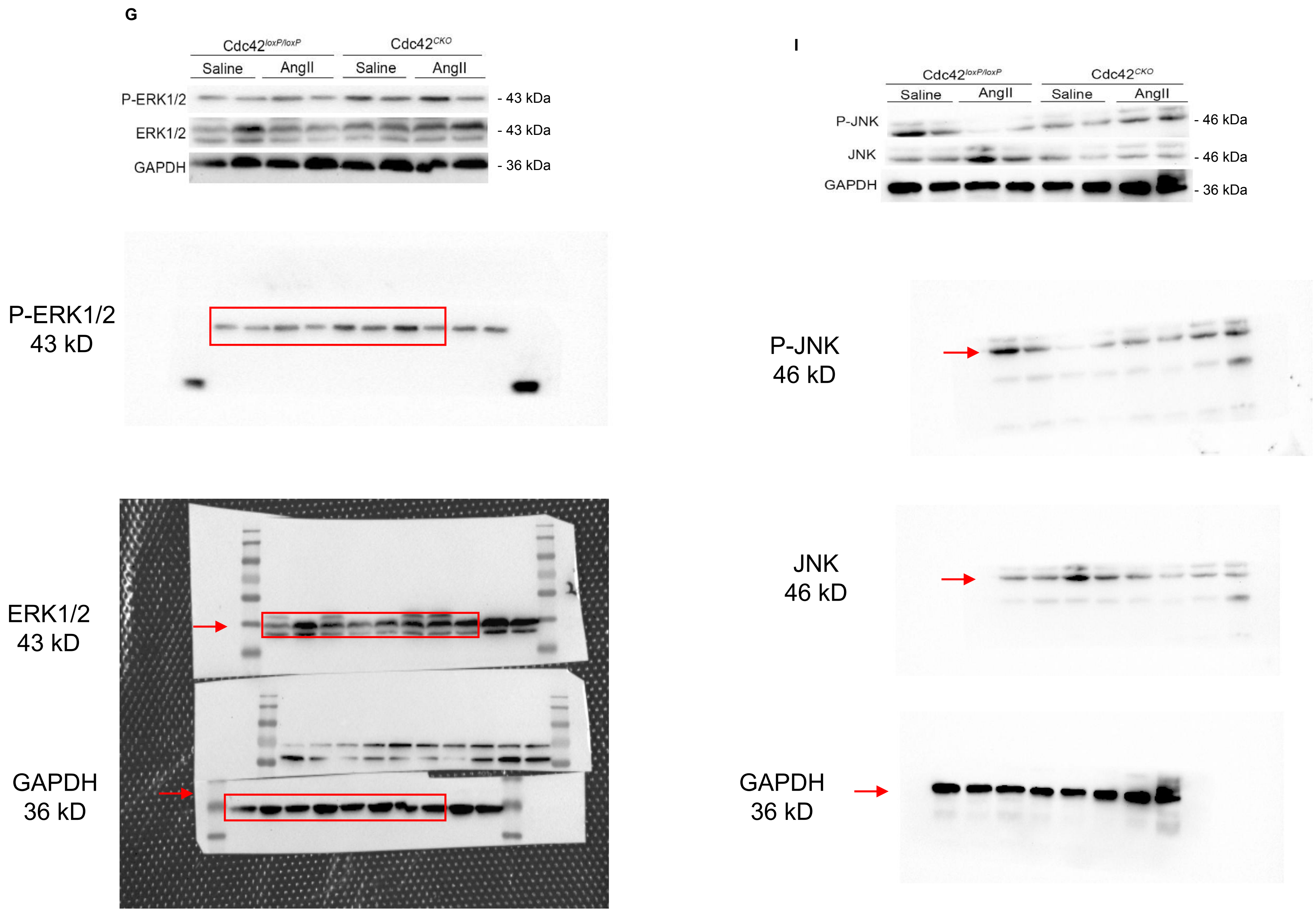

G
Cdc42 loxP/loxP
Cdc42 CKO
Saline
AngII
Saline
AngII
P-ERK1/2
ERK1/2
GAPDH
- 43 kDa
- 43 kDa
- 36 kDa
I
Cdc42 loxP/loxP
Cdc42 CKO
Saline
AngII
Saline
AngII
P-JNK
JNK
GAPDH
- 46 kDa
- 46 kDa
- 36 kDa
P-ERK1/2
43 kD
ERK1/2
43 kD
GAPDH
36 kD
P-JNK
46 kD
JNK
46 kD
GAPDH
36 kD

Figure 2

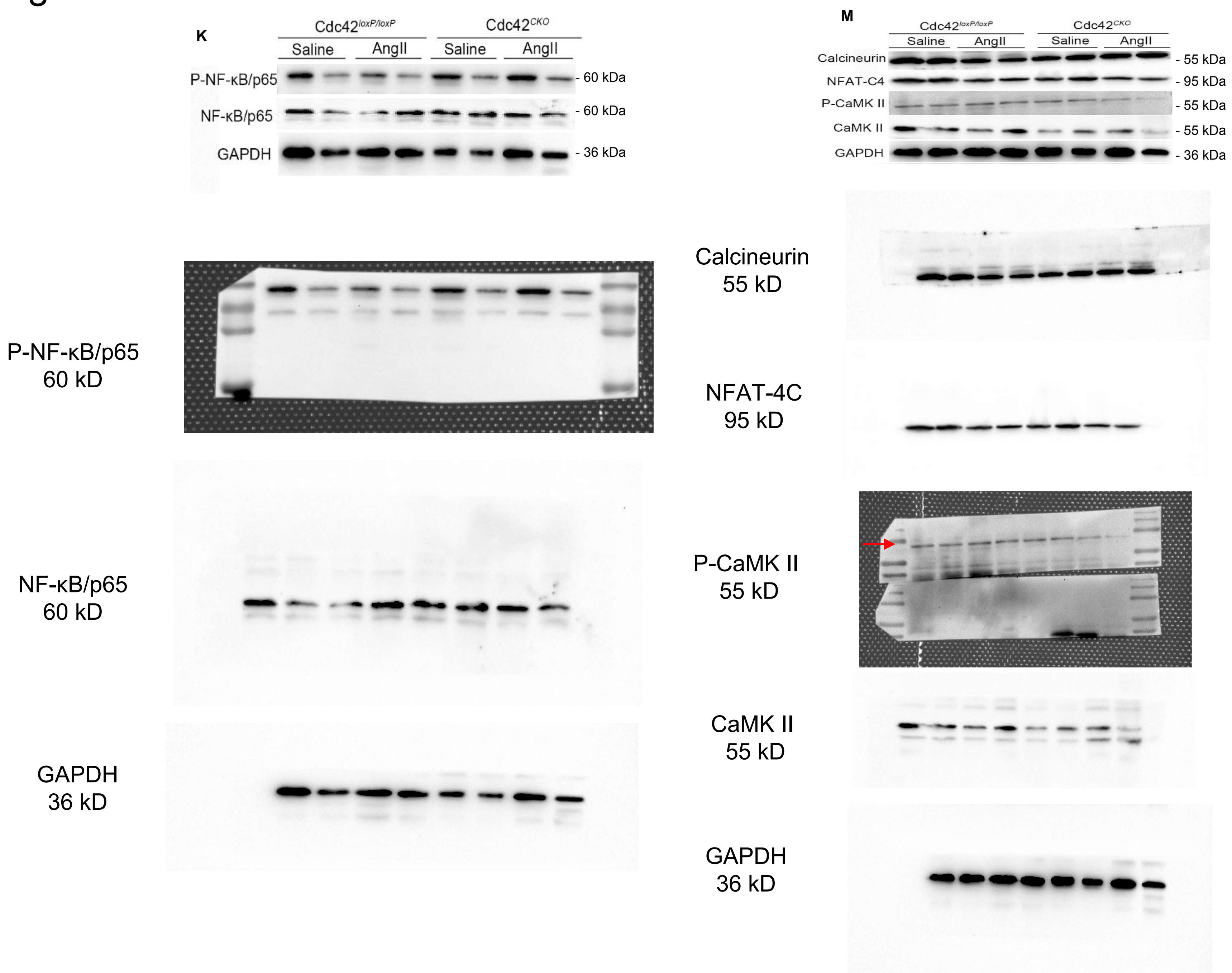
K
Cdc42loxP/loxP
Cdc42CKO
Saline
AngII
Saline
AngII
P-NF-κB/p65
NF-κB/p65
GAPDH
- 60 kDa
- 60 kDa
- 36 kDa
M
Cdc42loxP/loxP
Cdc42CKO
Saline
AngII
Saline
AngII
Calcineurin
NFAT-C4
P-CaMK II
CaMK II
GAPDH
- 55 kDa
- 95 kDa
- 55 kDa
- 55 kDa
- 36 kDa
P-NF-κB/p65
60 kD
NF-κB/p65
60 kD
GAPDH
36 kD
Calcineurin
55 kD
NFAT-4C
95 kD
P-CaMK II
55 kD
CaMK II
55 kD
GAPDH
36 kD

Figure 3

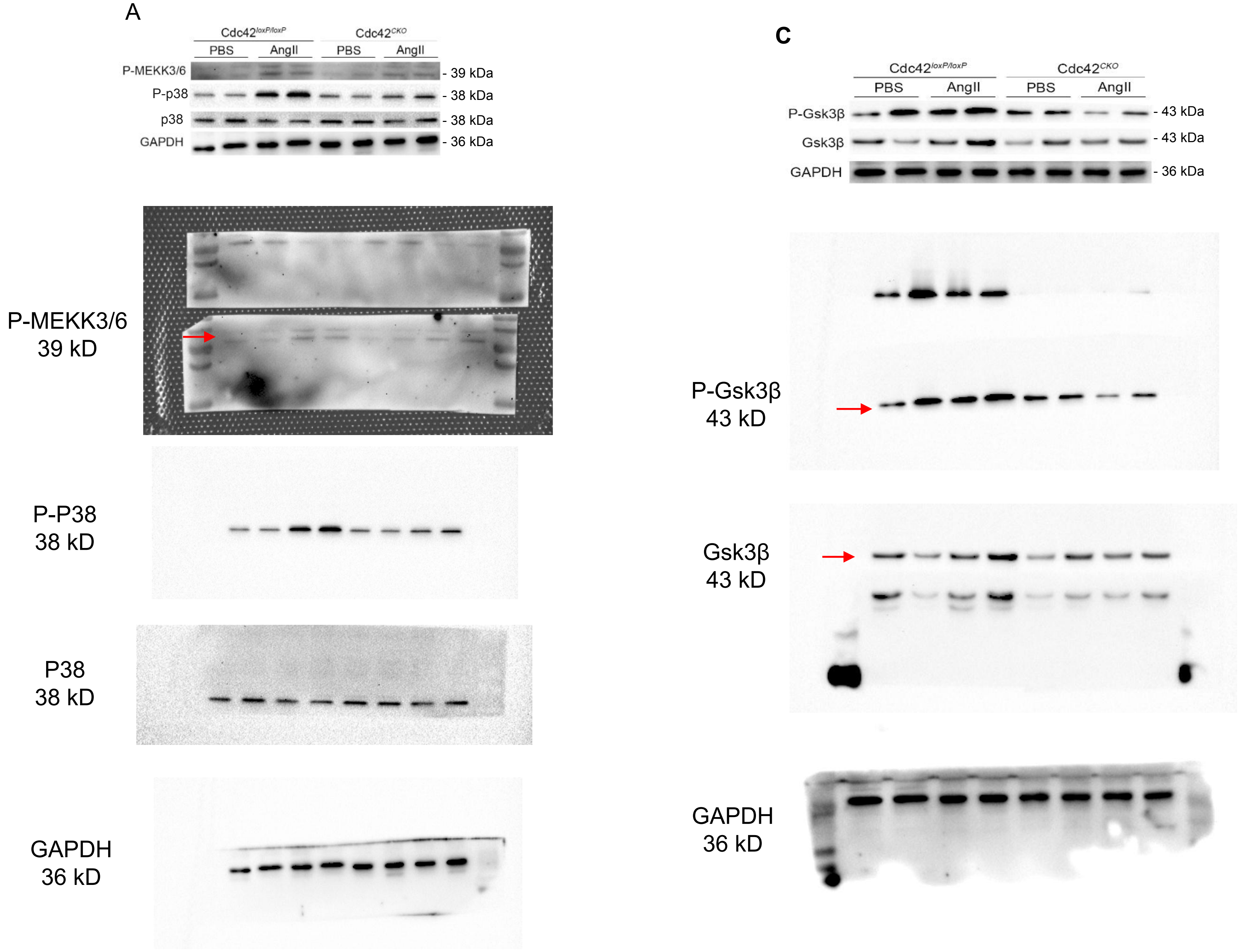
A
Cdc42loxP/loxP
Cdc42CKO
PBS
AngII
PBS
AngII
P-MEKK3/6
P-p38
p38
GAPDH
- 39 kDa
- 38 kDa
- 38 kDa
- 36 kDa
P-MEKK3/6
39 kD
P-P38
38 kD
P38
38 kD
GAPDH
36 kD
C
Cdc42loxP/loxP
Cdc42CKO
PBS
AngII
PBS
AngII
P-Gsk3β
Gsk3β
GAPDH
- 43 kDa
- 43 kDa
- 36 kDa
P-Gsk3β
43 kD
Gsk3β
43 kD
GAPDH
36 kD

Figure 3

E

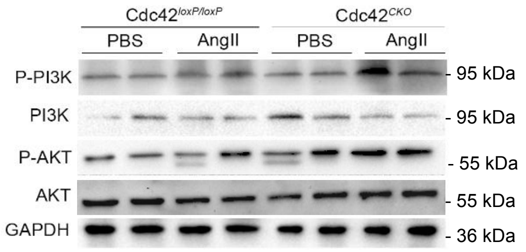

P-PI3K
95 kD

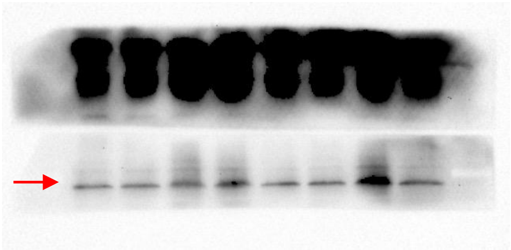

P-AKT
55 kD

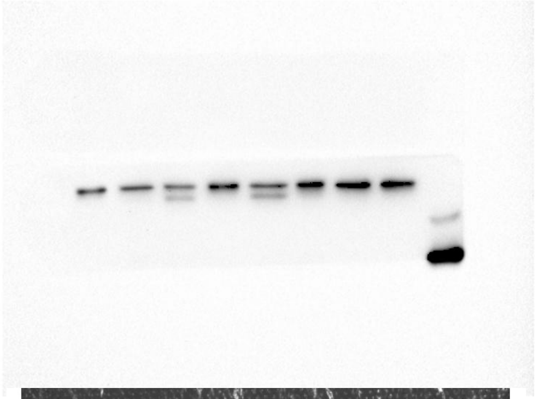

AKT
55 kD

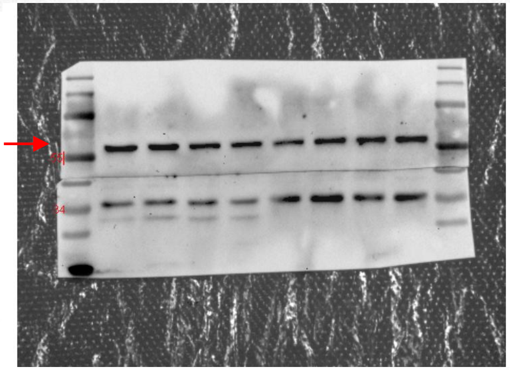

PI3K
95 kD

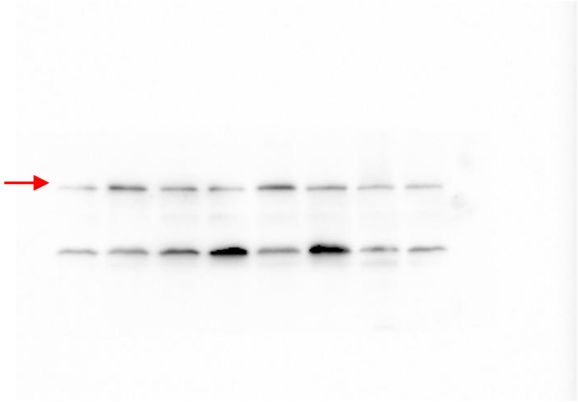

GAPDH
36 kD

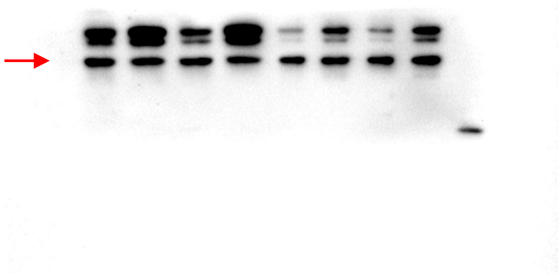

Figure 3

G

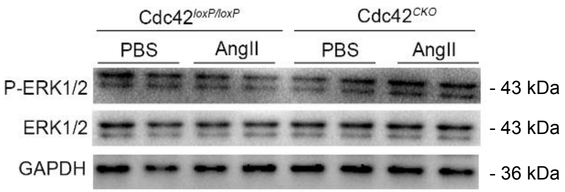

P-ERK1/2
43 kD

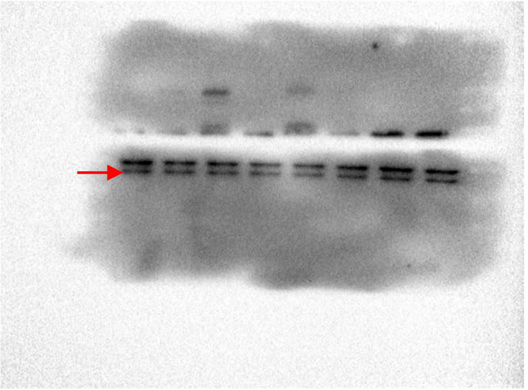

ERK1/2
43 kD

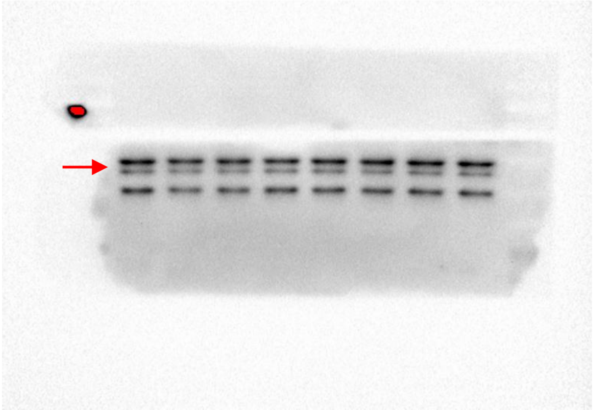

GAPDH
36 kD

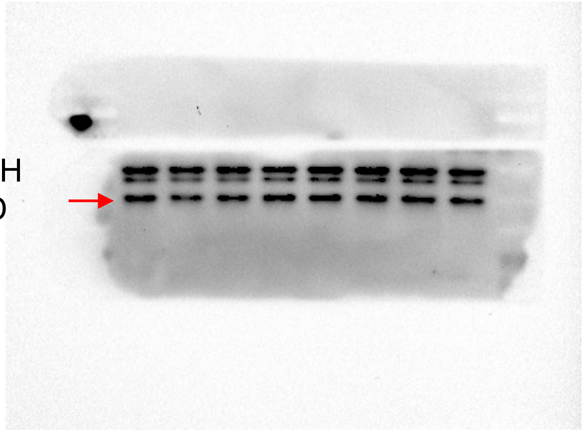

Figure 3

I

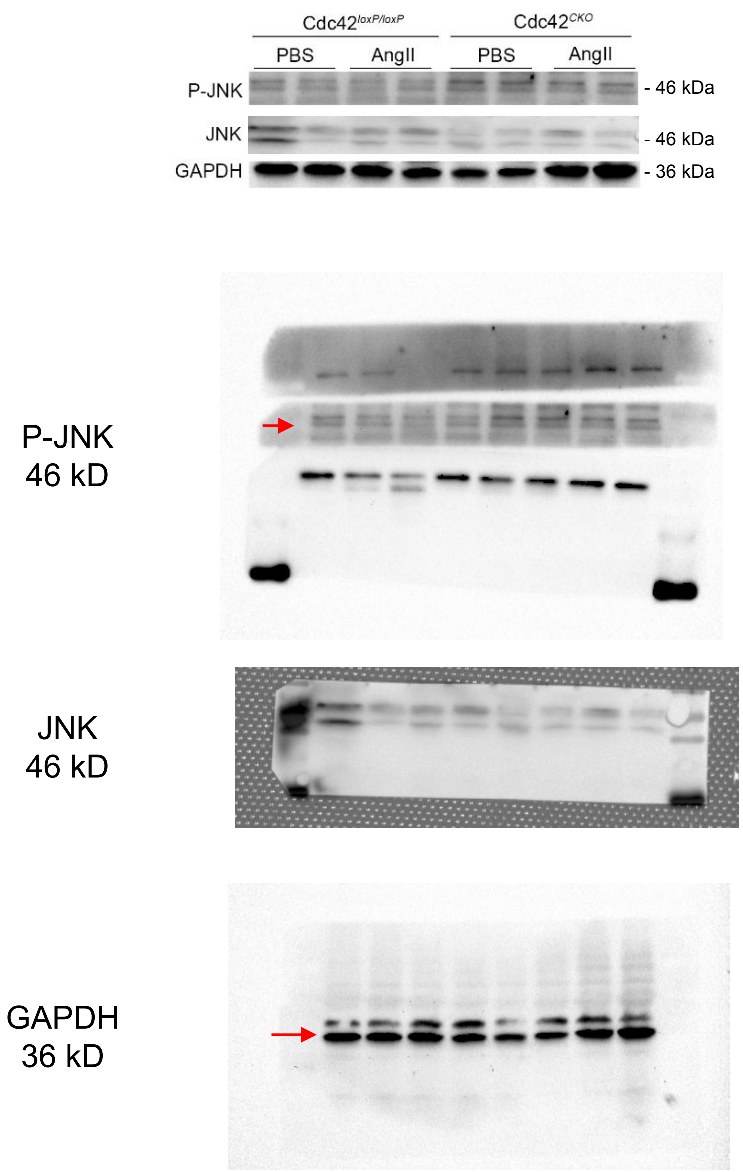
Cdc42$^{loxP/loxP}$
Cdc42^{CKO}
PBS
AngII
PBS
AngII
P-JNK
- 46 kDa
JNK
- 46 kDa
GAPDH
- 36 kDa
P-JNK
46 kD
JNK
46 kD
GAPDH
36 kD

K

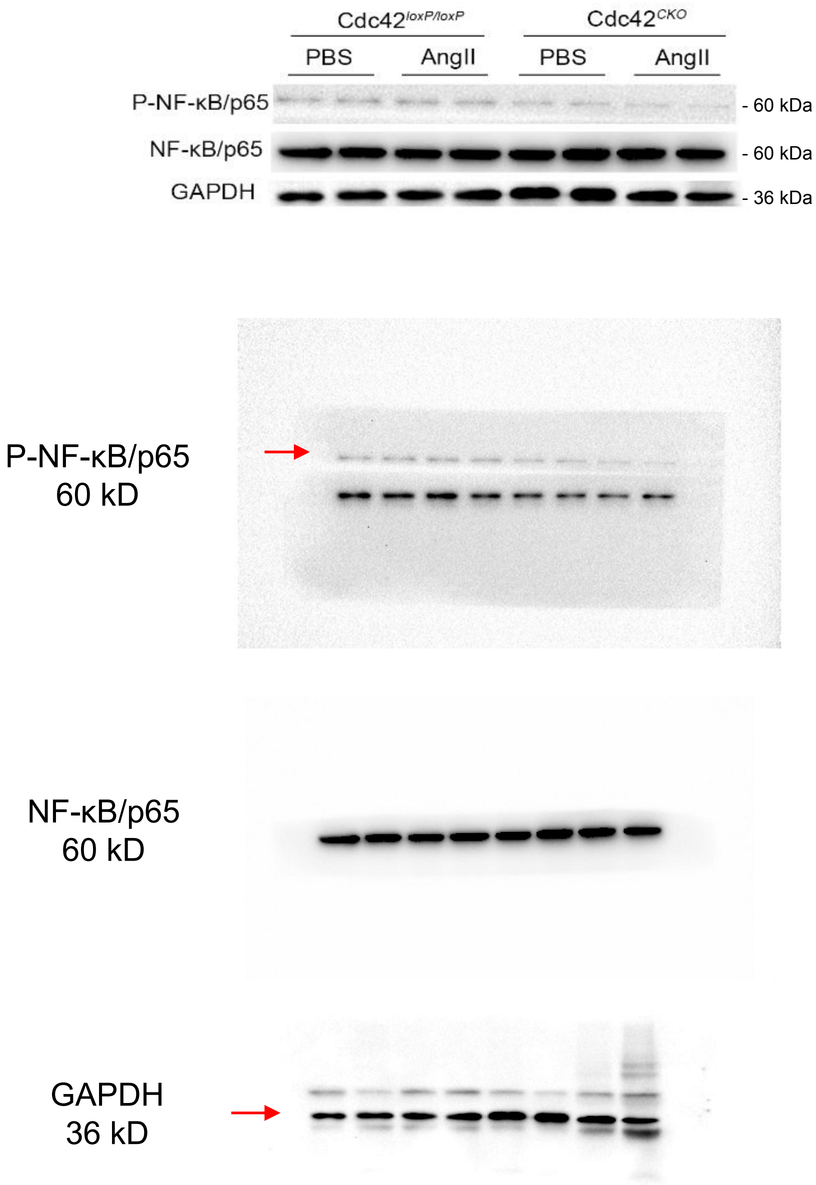
Cdc42$^{loxP/loxP}$
Cdc42^{CKO}
PBS
AngII
PBS
AngII
P-NF-κB/p65
- 60 kDa
NF-κB/p65
- 60 kDa
GAPDH
- 36 kDa
P-NF-κB/p65
60 kD
NF-κB/p65
60 kD
GAPDH
36 kD

Figure 3

M

M

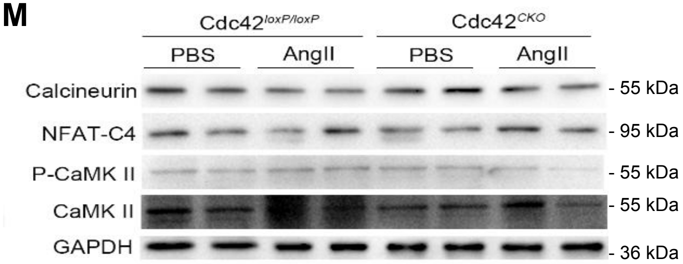

Calcineurin
55 kD

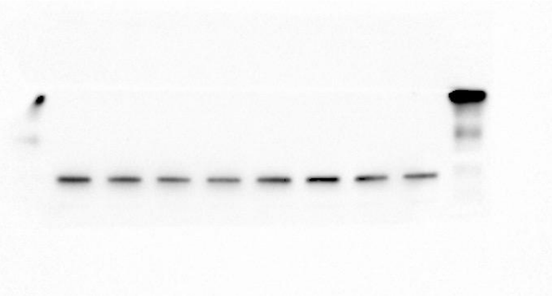

P-CaMK II
55 kD

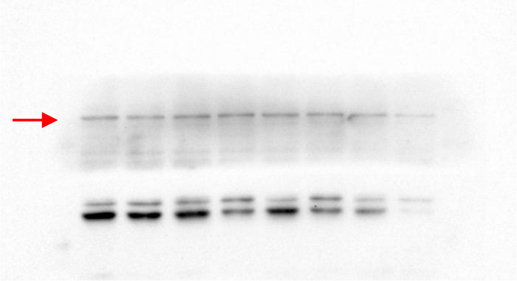

CaMK II
55 kD

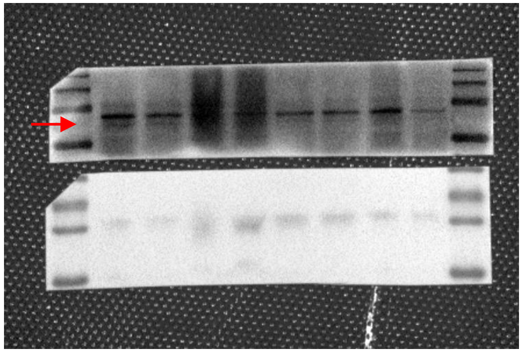

NFAT-4C
95 kD

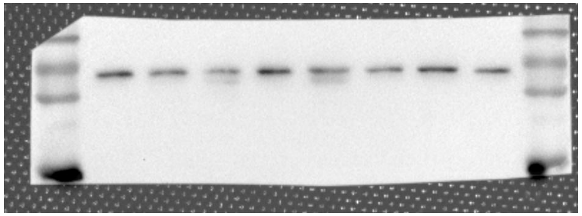

GAPDH
36 kD

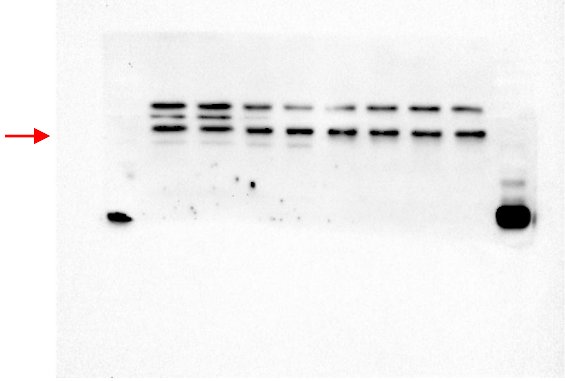

Figure 4

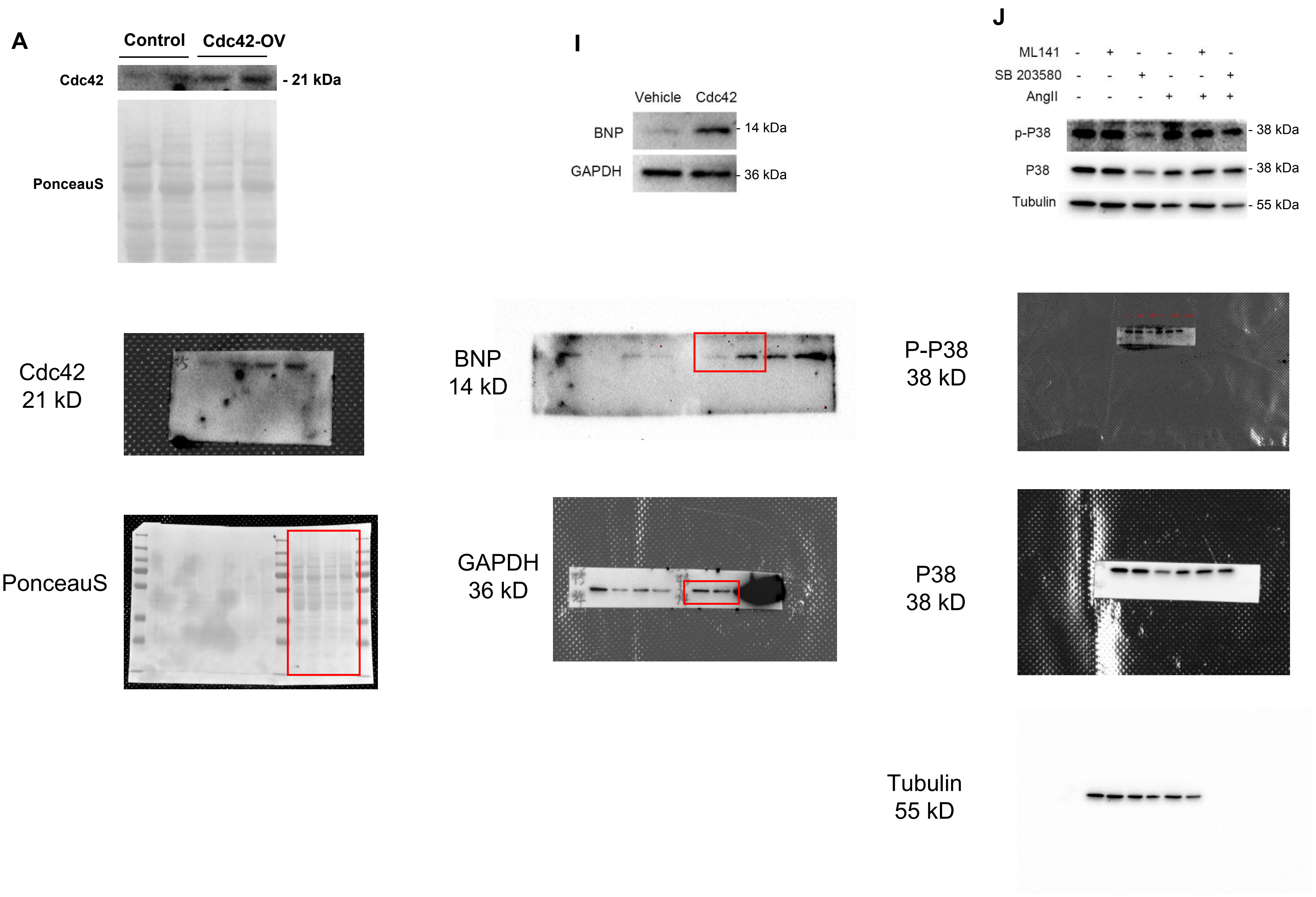
A
Control
Cdc42-OV
Cdc42
- 21 kDa
PonceauS
I
Vehicle
Cdc42
BNP
- 14 kDa
GAPDH
- 36 kDa
J
ML141 - + - - + -
SB 203580 - - + - - +
AngII - - - + + +
p-P38
- 38 kDa
P38
- 38 kDa
Tubulin
- 55 kDa
Cdc42
21 kD
PonceauS
BNP
14 kD
GAPDH
36 kD
P-P38
38 kD
P38
38 kD
Tubulin
55 kD

Figure 6

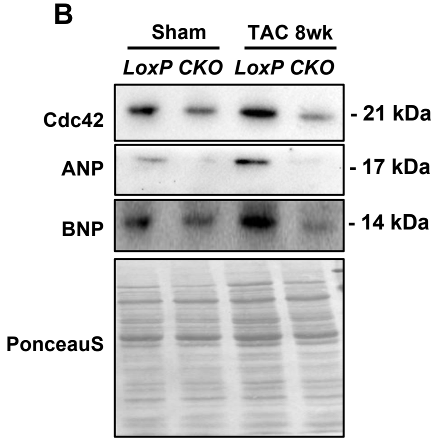

B

Cdc42
21 kD

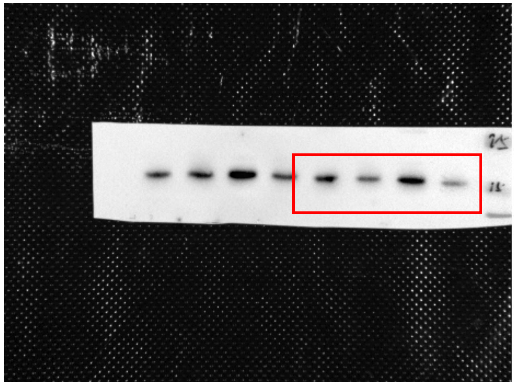

BNP
14 kD

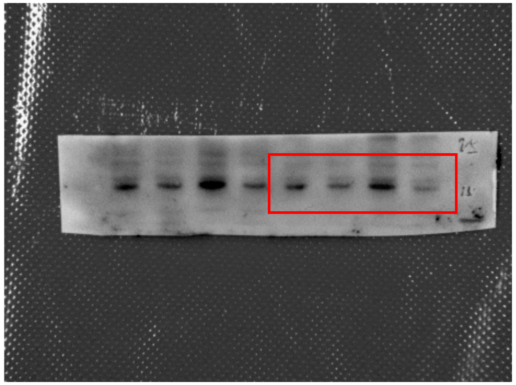

ANP
17 kD

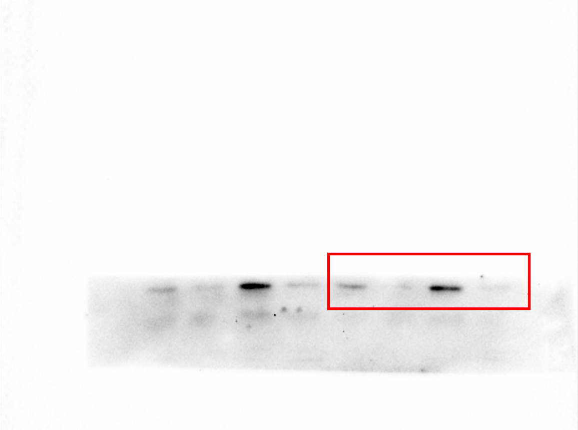

PonceauS

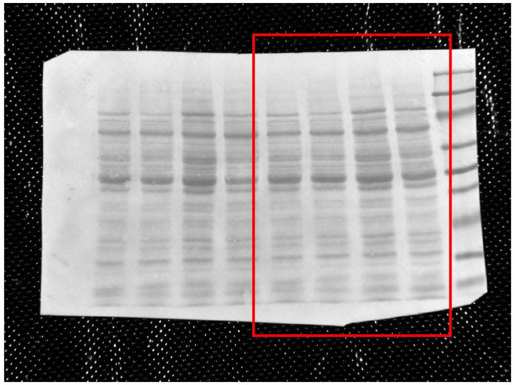

Figure 6

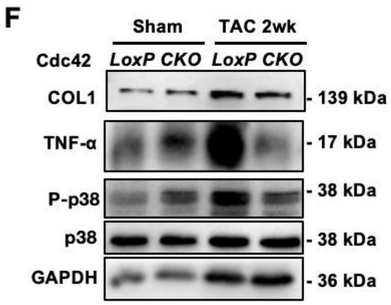

F

COL1
139 kD

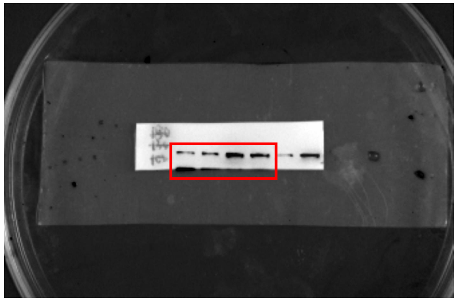

GAPDH
36 kD

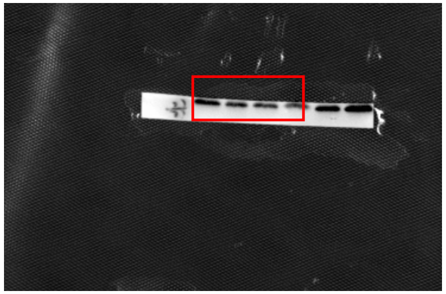

This figure displays p-AKT and AKT loading controls, but was not included in the manuscript.

TNF-α
17 kD

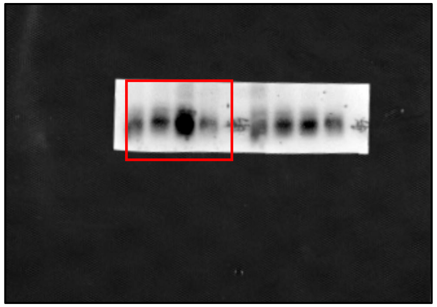

P38
38 kD

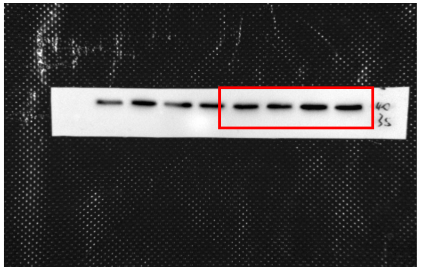

GAPDH
36 kD

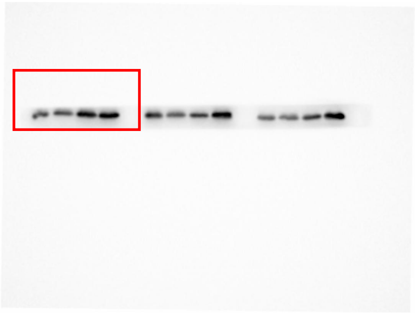

P-P38
38 kD

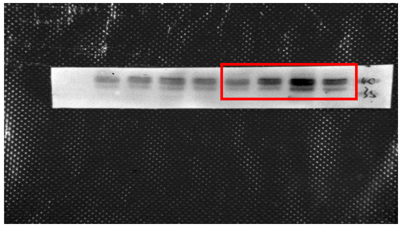

PonceauS

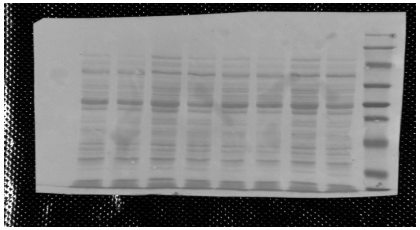

Figure 7

A

IL-6
17kDa

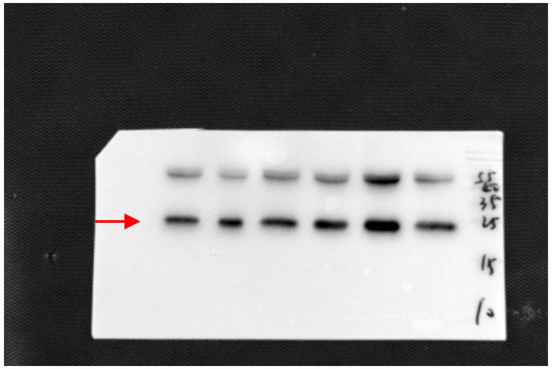

TNF-α
17kDa

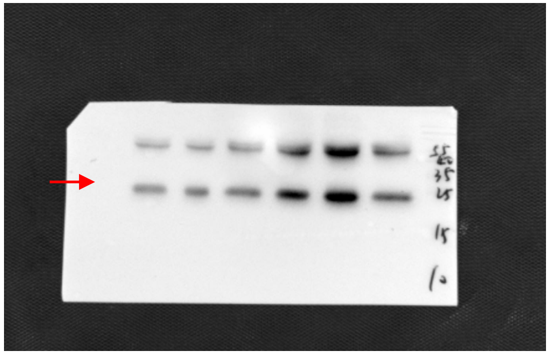

Ponceau S

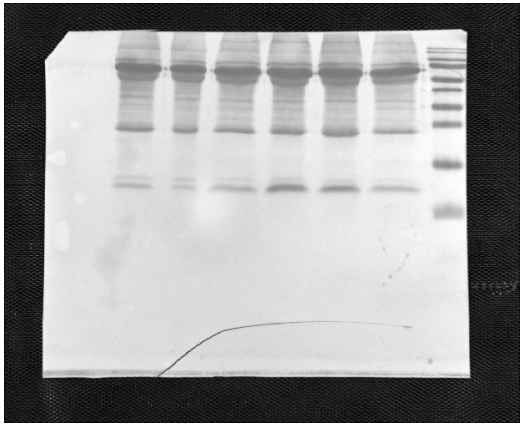

A

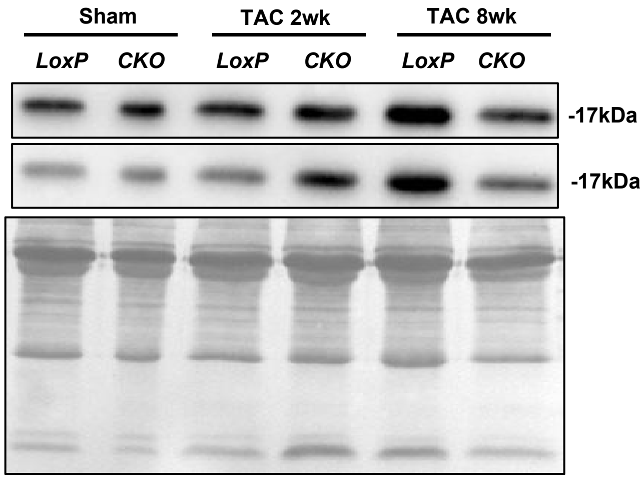

Supplement: Supplementary file 2 — Supplementary Material 3 [file 18_2025_5743_MOESM2_ESM.pdf]
